# IGF-1Eb Isoform Immunoreactivity May Be Associated with Placental Dysfunction and Vascular Pathology in Fetal Growth Restriction (FGR)

**DOI:** 10.3390/biom16091281

**Published:** 2026-09-04

**Authors:** Apostolos Fasoulopoulos, Michail Varras, Fani-Niki Varra, Viktoria-Konstantina Varra, Anastassios Philippou, Alexandros Gryparis, Argyro Papadopetraki, Petros Stellatos, Athina Pesiridou, Kleopatra Paparizou, Anastasia Evangelia Konstantinidou

**Affiliations:** 1 Fourth Obstetrics and Gynecology Department, ‘Elena Venizelou’ General Hospital of Athens, 11521 Athens, Greece; fasoulap1@gmail.com (A.F.); petstellatos@gmail.com (P.S.); pesiridouath@gmail.com (A.P.); 2 Medical School, Democritus University of Thrace, 68100 Alexandroupolis, Greece; 3 Department of Physiology, Medical School, National Kapodistrian University of Athens, 11527 Athens, Greece; tfilipou@med.uoa.gr (A.P.); arg.papadopetraki@gmail.com (A.P.); 4 Department of Pharmacy, School of Health Sciences, University of Patras, University Campus, 26504 Patra, Greece; 5 Advanced Aesthetics and Cosmetic Science, Development-Quality Control and Safety of New Cosmetic Products, Division of Aesthetics and Cosmetic Science, Department of Biomedical Sciences, School of Health and Welfare Sciences, University of West Attica, Aigaleo, 12243 Athens, Greece; 6 Department of Speech and Language Therapy, University of Ioannina, 45500 Ioannina, Greece; al.grip@gmail.com; 7 Unit of Pediatric-Perinatal Pathology, First Department of Pathology, Medical School, National and Kapodistrian University of Athens, 11527 Athens, Greece; kleo.paparizou@gmail.com (K.P.); ankon@med.uoa.gr (A.E.K.); 8 Department of Pathology, Aretaieion University Hospital, Medical School, National and Kapodistrian University of Athens, 11528 Athens, Greece

**Keywords:** fetal growth restriction (FGR), insulin-like growth factor-1 (IGF-1), IGF-1Eb isoform, placental insufficiency, syncytiotrophoblast, placental vascular pathology, spiral uterine artery remodeling, uteroplacental vascular remodeling, immunohistochemistry, maternal vascular malperfusion (MVM)

## Abstract

Background: Fetal growth restriction (FGR) is associated with placental dysfunction and adverse perinatal outcomes. Although insulin-like growth factor-1 (IGF-1) signaling is important for placental and fetal development, the mRNA expression, immunopositivity and potential biological significance of specific IGF-1 isoforms in FGR remain incompletely characterized. This study investigated placental IGF-1Eb mRNA expression and immunopositivity in FGR pregnancies compared with appropriate-for-gestational-age (AGA) pregnancies. Methods: A total of 62 third-trimester human placentas were analyzed, including 47 from pregnancies complicated by FGR and 15 from pregnancies with AGA fetal growth. The mRNA expression of the IGF-1Eb isoform was assessed by reverse-transcription quantitative PCR in a subset of 28 fresh placental samples. IGF-1Eb protein immunoreactivity was assessed in paraffin-embedded tissue sections. Histopathological lesions were classified according to the Amsterdam criteria, and associations with clinical, demographic, and pathological parameters were evaluated using appropriate statistical analyses. Results: IGF-1Eb mRNA expression did not differ significantly between the FGR and AGA groups. In contrast, significant differences in IGF-1Eb immunoreactivity were observed in selected placental compartments. Moderate immunoreactivity in the perivillous syncytiotrophoblast was more frequent in FGR placentas and was associated with histological features of maternal vascular malperfusion, as well as with gestational age, neonatal birth weight, placental weight, maternal body mass index, and fetal sex. Increased IGF-1Eb immunopositivity was also observed in the endothelium of maternal decidual and fetal villous vessels in FGR placentas. No significant differences were observed in IGF-1Eb immunoreactivity in the extravillous trophoblast. Conclusions: In this observational study, placental IGF-1Eb protein immunoreactivity, but not mRNA expression, differed between FGR and AGA pregnancies in selected placental compartments. These findings indicate compartment-specific differences in IGF-1Eb protein immunoreactivity, associated with FGR and placental pathological features. However, the observational and cross-sectional design does not establish causality or a functional role for IGF-1Eb in placental dysfunction. Larger prospective studies incorporating functional validation are required to clarify the biological significance of these findings.

## 1. Introduction

Fetal growth restriction (FGR) is a significant obstetric disorder, generally defined by an estimated fetal weight below the 10th percentile relative to gestational age on prenatal ultrasonography and arising from diverse etiologies, including placental insufficiency, maternal disorders, and fetal abnormalities [1,2]. This condition is associated with a higher risk of perinatal morbidity and mortality and may predispose affected individuals to adverse health outcomes later in life, particularly metabolic, renal, and cardiovascular complications [3,4]. Pathophysiologically, FGR refers to impaired fetal growth resulting in failure to reach the genetically determined growth potential, primarily as a consequence of abnormal angiogenesis and insufficient transfer of oxygen and nutrients during critical stages of fetal growth, particularly in the third trimester of pregnancy [5,6]. FGR may occur with no identifiable or known medical cause, after ruling out all common underlying risk factors. Despite extensive investigation, the pathophysiological mechanisms underlying FGR are not fully understood [7].

Among its components, insulin-like growth factor-1 (IGF-1) is a key regulator of trophoblast proliferation, survival, and placental vascularization [8,9,10]. The IGF-1 gene undergoes alternative splicing, resulting in the production of multiple isoforms with distinct E-peptide extensions, including IGF-1Ea, IGF-1Eb, and IGF-1Ec, which may exert specific autocrine and paracrine effects [11,12,13]. The IGF-1Eb isoform has been implicated in biological mechanisms related to tissue growth, regeneration, and adaptive responses to stress, and growing evidence also links IGF signaling to the pathophysiology of gynecological disorders, including endometriosis, endometrial carcinoma, and uterine leiomyomas [11,12,14,15,16]. However, its mRNA expression and immunopositivity patterns and its functional relevance within the placenta remain insufficiently characterized [13]. Given the importance of IGF-1 signaling during fetal development, dysregulation of individual IGF-1 isoforms may play a role in placental dysfunction and contribute to the pathogenesis of FGR [13]. Characterizing isoform-specific mRNA expression and immunoreactivity patterns may therefore provide valuable insights into disease mechanisms and identify candidate targets for future biomarker studies of placental dysfunction.

In continuance of our previous research on the IGF-1Ea isoform [13], this study seeks to characterize IGF-1Eb isoform mRNA expression and immunoreactivity patterns in placental tissue from singleton pregnancies with normal fetal growth or FGR during the third trimester. Furthermore, we seek to explore potential correlations of placental IGF-1Eb mRNA expression or/and immunoreactivity with relevant clinical outcomes as well as histopathological parameters. Elucidating these relationships may enhance our understanding of the molecular alterations associated with FGR and provide a basis for future studies investigating potential diagnostic and therapeutic applications.

## 2. Material and Methods

### 2.1. Patient Population

This study included 62 placental samples obtained from singleton pregnancies, consisting of 15 appropriate-for-gestational-age (AGA) pregnancies and 47 pregnancies complicated by fetal growth restriction (FGR), all previously analyzed for IGF-1Ea mRNA expression and immunoreactivity [13]. Deliveries were performed either vaginally or by cesarean section at the General Maternity Hospital of Athens “Elena Venizelou” (Athens, Greece).

Maternal age ranged from 16 to 46 years, and gestational age at delivery ranged from 26 to 41 weeks. Written informed consent was obtained from all participants. The study protocol was approved by the Scientific Committee of the General Maternity Hospital of Athens “Elena Venizelou” (approval no. 2nd Scientific Committee Meeting/8th agenda/23 January 2018) and the Research and Bioethics Committee of the Medical School of the National and Kapodistrian University of Athens (approval no. 1718016683).

Inclusion criteria for the FGR group consisted of pregnancies with an ultrasound-estimated fetal weight (EFW) below the 5th percentile for gestational age, selected to identify pregnancies with more severe fetal growth restriction. The 5th percentile was used as an inclusion threshold for cohort selection rather than as a distinct diagnostic definition of FGR. Doppler parameters and specific markers of placental dysfunction were not systematically recorded in the original cohort and were therefore not incorporated into the classification of fetal growth restriction. FGR cases associated with preeclampsia were also included. Control placentas were derived from uncomplicated AGA pregnancies delivering healthy neonates with birth weights between the 10th and 90th percentiles. All control cases were full-term (>37 weeks of gestation) and showed no gross pathological abnormalities. The maternal medical history and pregnancy records were reviewed to identify conditions and treatments that could potentially affect placental development or function. Pregnancies complicated by pre-existing hypertension, pregestational diabetes, abnormal glucose tolerance during pregnancy, major hepatic, cardiovascular, renal, or endocrine disorders, or other clinically significant maternal conditions were excluded. Maternal autoimmune disorders, including systemic lupus erythematosus (SLE), were also considered exclusion criteria.

Gestational age was calculated from the reported date of the last menstrual period and subsequently verified using first-trimester crown–rump length measurements. For each participant, relevant maternal and neonatal characteristics were collected, including maternal age and BMI, gestational age at birth, neonatal and placental weights, and fetal sex.

The maternal, pregnancy, and neonatal characteristics of the pregnancies included in the present placental mRNA analysis are summarized in Table 1. The molecular analysis comprised placental samples from 28 pregnancies, including 13 AGA pregnancies and 15 FGR pregnancies. Maternal age, gestational age at delivery, neonatal birth weight, placental weight, maternal body mass index (BMI), and fetal sex were considered in the characterization of the study population. The categorical distribution of these parameters within the subgroup analyzed for mRNA expression is presented in Table 1. The broader clinical and clinicopathological characteristics of the original cohort have been reported previously [13].

### 2.2. Tissue Sampling

The sampling methodology was identical to that previously described for IGF-1Ea analysis [13]. Placental specimens were collected between February 2018 and August 2021, with all tissues obtained within 15 min following delivery. After removal of the fetal membranes and umbilical cord, placentas were weighed prior to further processing.

For RNA analysis, fresh placental tissue was obtained from 28 cases. Samples were excised from central placental regions located approximately 5 cm from the umbilical cord insertion site, while peripheral areas were avoided. Villous tissue spanning from the decidua basalis to the fetal surface was selected, excluding regions exhibiting calcification, infarction, extensive fibrin accumulation, or intervillous thrombosis. Tissue specimens were dissected into small pieces, washed with 0.9% phosphate-buffered saline to eliminate residual blood, rapidly frozen, and stored at −80 °C until RNA isolation. The remaining placental specimens were preserved in 10% buffered formalin at room temperature for seven days before undergoing histological and immunohistochemical processing.

### 2.3. Histopathology

Formalin-fixed, paraffin-embedded tissue samples were processed using an automated tissue processor (Donatello, Diapath, Bergamo, Italy EU, Ottix Shaper & Ottix Plus) and subsequently stained with hematoxylin (Biognost Ltd. Medjugorska 59, 10040, Zagreb, Croatia EU) for 5 min, followed by 1% alcoholic eosin Y for 10 s, with all staining procedures performed at room temperature.

Histopathological evaluation and lesion classification were performed according to the Amsterdam criteria [17]. Placental findings were assessed for major patterns including: (i) maternal vascular malperfusion (MVM), (ii) fetal vascular malperfusion (FVM), (iii) massive perivillous fibrin/fibrinoid deposition (MPFD), (iv) inflammatory lesions such as chronic villitis of unknown etiology (VUE), and (v) delayed villous maturation (DVM). Additional miscellaneous lesions were also recorded when present.

### 2.4. RNA Isolation and cDNA Synthesis

Frozen placental tissues derived from 13 AGA and 15 FGR complicated third-trimester pregnancies were included, with all samples having undergone prior analysis of IGF-1Ea expression [13]. Total RNA was isolated from the frozen placental tissue using TRItidy G™ reagent (PanReac AppliChem GmbH, Darmstadt, Germany) following the manufacturer’s recommended protocol. Placental tissue samples were sectioned into approximately 12 × 8 mm fragments and homogenized in 0.5 mL of TRItidy G reagent. After the addition of 500 µL isopropanol, samples were centrifuged at 13,226× *g* for 15 min at room temperature. The resulting RNA pellet was washed with 75% ethanol and subsequently resuspended in 20 µL diethylpyrocarbonate-treated water. Complementary DNA (cDNA) synthesis was carried out by reverse transcription using the ProtoScript^®^ II First Strand cDNA Synthesis Kit (New England BioLabs, Ipswich, MA, USA; cat. no. E6560L) according to the manufacturer’s instructions.

### 2.5. RNA Quality Control

The quality and purity of the extracted total RNA were assessed spectrophotometrically using a NanoDrop 2000 spectrophotometer (Thermo Scientific, Waltham, MA, USA) by measuring absorbance at 260 and 280 nm. Samples with a 260/280 ratio between 1.8 and 2.0 were considered to have adequate RNA purity. RNA integrity was further assessed by electrophoresis on 1% agarose gels, followed by visualization under ultraviolet (UV) light after ethidium bromide staining. The integrity of the RNA was confirmed by visualization of the 18S and 28S ribosomal RNA bands. To account for potential variations in the amount of starting material and reverse-transcription efficiency, 18S ribosomal RNA was used as the endogenous control for normalization of mRNA expression.

### 2.6. Reverse Transcriptase Quantitative PCR (RT-qPCR)

The oligonucleotide primer sequences used for IGF-1Eb amplification were forward primer, 5′-ATGTCCTCCTCGCATCTCT-3′, and IGF-1Eb reverse primer, 5′-CCTCCTTCTGTTCCCCTC-3′, generating a 411 bp amplicon. Quantitative PCR analysis was performed using the Bio-Rad iCycler IQ5 Multicolor Real-Time PCR Detection System (Bio-Rad Laboratories, Inc., Hercules, CA, USA). Each reaction mixture contained 12.5 µL iQ™ SYBR Green Supermix (Bio-Rad Laboratories, Inc., Hercules, CA, USA), 50 ng cDNA template, and 0.4 µM of each primer, with ddH_2_O added to achieve a final reaction volume of 20 µL. Negative controls lacking template DNA were included in every run to confirm the absence of contamination. The amplification protocol consisted of an initial denaturation step at 95 °C for 4 min, followed by 45 cycles of denaturation at 95 °C for 12 s, annealing at 61 °C for 30 s, and extension at 72 °C for 30 s, with a final extension step at 72 °C for 5 min. Relative gene expression was calculated using the 2^−ΔΔCt^ method, with 18S ribosomal RNA used for normalization between FGR and control placental samples [18]. All reactions were performed in duplicate.

### 2.7. Immunohistochemistry

Immunohistochemical analysis was performed on formalin-fixed, paraffin-embedded placental specimens from 15 AGA and 47 FGR pregnancies, using the same methodological protocol previously applied for IGF-1Ea assessment [13]. Staining was performed using the EnVision FLEX+ Mouse High pH (Link) system (cat. no. K8002; Dako, Agilent Technologies, Carpinteria, CA, USA). Sections of 4 µm thickness were cut using a microtome, dried overnight at 37 °C, deparaffinized in xylene, and rehydrated through graded ethanol solutions. For antigen retrieval, the tissue sections were heated to 97 °C for 20 min in a PT module containing DAKO high-pH buffer (pH 9), and then allowed to cool to room temperature. Endogenous peroxidase activity was blocked by incubating the sections with 3% hydrogen peroxide for 5 min at room temperature under dark conditions. Following rinsing with distilled water and DAKO wash buffer, the tissue sections were incubated overnight at 4 °C with a polyclonal rabbit anti-human IGF-1Eb antibody diluted 1:500 in DAKO antibody diluent. This antibody is a custom-made anti-human IGF-1Eb isoform-specific antibody raised in rabbits and with its epitope located in the IGF-1Eb-specific part of the IGF-1 gene to detect only this specific isoform and ensuring no reactivity with the other IGF-1 isoforms, based on the amino acid sequence of the IGF-1Eb isoform, as previously described by our group [17]. A recent publication has confirmed the specificity of this antibody using immunofluorescence analysis and knockdown validation [19]. Following the washing step, the sections were incubated with EnVision FLEX+ Rabbit linker (cat. no. K8009; Dako, Agilent Technologies, Carpinteria, CA, USA) for 15 min at room temperature and subsequently rinsed again. The EnVision polymer was subsequently applied for 30 min, followed by another washing step. Signal detection was then performed using 3,3′-diaminobenzidine (DAB) for 10 min. Counterstaining was performed with hematoxylin for 5 min at room temperature. The tissue sections were subsequently dehydrated using increasing concentrations of ethanol and xylene before being mounted with a dibutyl phthalate–xylene mounting medium. Archived adrenal carcinoma tissue from surgical pathology specimens at Aretaieion University Hospital, Medical School, National and Kapodistrian University of Athens, was used as the positive control for IGF-1Eb immunohistochemical staining. Negative controls were processed identically, except for omission of the primary antibody. Immunohistochemical assessment was initially performed under light microscopy at 100× magnification. For each case, five randomly selected fields were evaluated, and semi-quantitative analysis was carried out at 200× magnification using a grid system. Two experienced pathologists independently assessed all specimens, with the final score for each sample determined by consensus. Both pathologists assessed each slide independently and a single consensus score was then agreed and recorded for each sample; the individual pre-consensus scores were not retained in the study database, and a formal measure of interobserver reproducibility could therefore not be derived (see Limitations).

#### 2.7.1. Assessment of Immunostaining in the Perivillous Trophoblast

The methodology applied was consistent with that previously used for IGF-1Ea evaluation [13]. Specifically, a semi-quantitative scoring system was employed to assess IGF-1Eb immunolabeling in the perivillous syncytiotrophoblast. Specimens were classified according to the proportion of syncytial areas exhibiting positive immunostaining: a score of 0 represented 0–10% positivity (negative), a score of 1 corresponded to 11–50% positivity (moderate immunoreactivity), and a score of 2 indicated ≥51% positive areas (high immunoreactivity). Immunostaining intensity was additionally assessed semi-quantitatively using a four-tier scoring system ranging from 0 to 3, representing absent, weak, moderate, and strong immunoreactivity, respectively.

#### 2.7.2. Assessment of Immunostaining in the Extravillous Trophoblast

The methodology used was identical to that applied for IGF-1Ea evaluation [13]. Extravillous trophoblastic cells were assessed for IGF-1Eb immunoreactivity and categorized as negative, indicating the absence of detectable staining, or positive, defined by the presence of stained cells. Staining intensity was also quantified semi-quantitatively on a four-point scale, with scores of 0, 1, 2, and 3 corresponding to absent, weak, moderate, and strong immunoreactivity, respectively.

#### 2.7.3. Assessment of Immunostaining in the Vascular Endothelium

The methodology followed was consistent with that previously applied for IGF-1Ea evaluation [13]. Immunoreactivity in the endothelial lining of maternal decidual vessels was evaluated and categorized as negative in the absence of staining or positive when at least one vessel within the basal plate exhibited detectable immunoreactivity. Likewise, endothelial staining in fetal villous vessels was examined and categorized as negative in the absence of immunoreactivity or positive when staining was detected in at least one fetal vessel.

### 2.8. Statistical Analysis

Categorical variables are summarized as counts and percentages, and continuous variables as medians with interquartile ranges. All results of the mRNA experiments are expressed as the mean of two independent experimental replicates ± standard deviation (SD). Associations between categorical variables were evaluated using Pearson’s chi-square test or Fisher’s exact test, as appropriate, and differences between continuous variables using the Mann–Whitney U test.

In addition to *p*-values, every comparison is accompanied by a measure of effect size with a 95% confidence interval: odds ratios obtained from the conditional maximum-likelihood estimator with exact confidence intervals, absolute risk differences with Newcombe score confidence intervals, Cramér’s V for contingency tables, and the rank-biserial correlation for rank-based comparisons. Absolute risk differences are given precedence over ratio measures in the interpretation of the findings, because with a control group of 15 placentas the odds-ratio scale is poorly determined and the upper limits of several confidence intervals are correspondingly extreme.

To examine whether the association between IGF-1Eb immunoreactivity and FGR persisted after accounting for the clinical variables that accompany it, multivariable models were fitted with each dichotomized IGF-1Eb endpoint as the dependent variable and study group as the exposure of interest. Because several strata were completely or quasi-completely separated, ordinary maximum likelihood is undefined or severely biased in these data; models were therefore estimated by Firth’s penalized likelihood, with penalized likelihood-ratio tests and profile penalized-likelihood confidence intervals.

Model 1 adjusted for gestational age at delivery and included all 62 placentas. Gestational age at delivery is not a conventional baseline confounder in this setting: in pregnancies complicated by FGR, it is itself influenced by disease severity and by clinical decisions regarding the timing of delivery. Furthermore, because appropriately grown preterm controls were not available, no preterm stratum contains an AGA placenta, and the adjusted estimates therefore depend on extrapolation across a region of the covariate distribution in which the two groups do not overlap. Adjustment for gestational age accordingly reduces, but does not eliminate, confounding by gestational age and by placental maturation, and cannot substitute for gestational-age-matched controls.

Model 2 additionally adjusted for maternal age and fetal sex and was restricted to the 40 placentas with complete covariate data. It was conducted as a sensitivity analysis, intended only to establish whether the direction and persistence of the associations were maintained when these covariates were added; because the data are sparse and fewer than two thirds of the cohort contribute, its adjusted odds ratios are not intended as quantitative effect estimates. Model 3 adjusted for gestational age and maternal vascular malperfusion.

Neonatal birth weight and placental weight were not entered as covariates. Both are closely related characteristics of the FGR phenotype itself rather than variables antecedent to it, and in an analysis in which FGR status is the exposure, they cannot function as conventional confounders. Maternal body mass index was examined in stratified analyses rather than in the primary models, because it was recorded for 32 of the 62 pregnancies and its inclusion would have discarded approximately half of the cohort. Preeclampsia status could not be established for every archived case and could therefore not be entered into any model; because preeclampsia may independently influence placental vascular pathology and molecular phenotype, all adjusted estimates should be read as reflecting adjustment for the available covariates only.

The subgroup analyses reported in Table 2, Table 3, Table 4, Table 5 and Table 6 are exploratory and were not pre-specified. To quantify the resulting multiplicity burden, *p*-values across all estimable subgroup comparisons were adjusted using the Benjamini–Hochberg false discovery rate procedure, and adjusted *q*-values are reported alongside the unadjusted *p*-values. Subgroups containing fewer than five placentas in either arm, and strata in which one arm contained no placentas, and no comparison is therefore estimable, are identified explicitly and are treated as hypothesis-generating only. For the RT-qPCR data, no a priori sample size calculation was performed; a post hoc precision analysis was therefore carried out to establish the smallest difference in transcript abundance that the available sample size could reliably have detected.

A *p*-value <0.05 (two-tailed) was considered statistically significant. Statistical analyses were performed using IBM SPSS software version 31.0 (IBM Corp., Armonk, NY, USA) and R version 4.5.3 (R Foundation for Statistical Computing, Vienna, Austria).

## 3. Results

### 3.1. Clinical and Pathological Findings

The clinicopathological features of the included cases were previously characterized and reported as part of the IGF-1Ea study [13]. The study group comprised 47 pregnancies complicated by fetal growth restriction FGR and 15 uncomplicated appropriate-for-gestational-age (AGA) pregnancies. The FGR group was defined on the basis of an ultrasound-estimated fetal weight below the fifth percentile for gestational age. As previously reported for this cohort [13], the FGR group included pregnancies associated with preeclampsia. Accordingly, the present analysis does not consider the cohort to represent a strictly idiopathic FGR population.

### 3.2. Placental IGF-1Eb Transcript Expression

**Figure 1:**
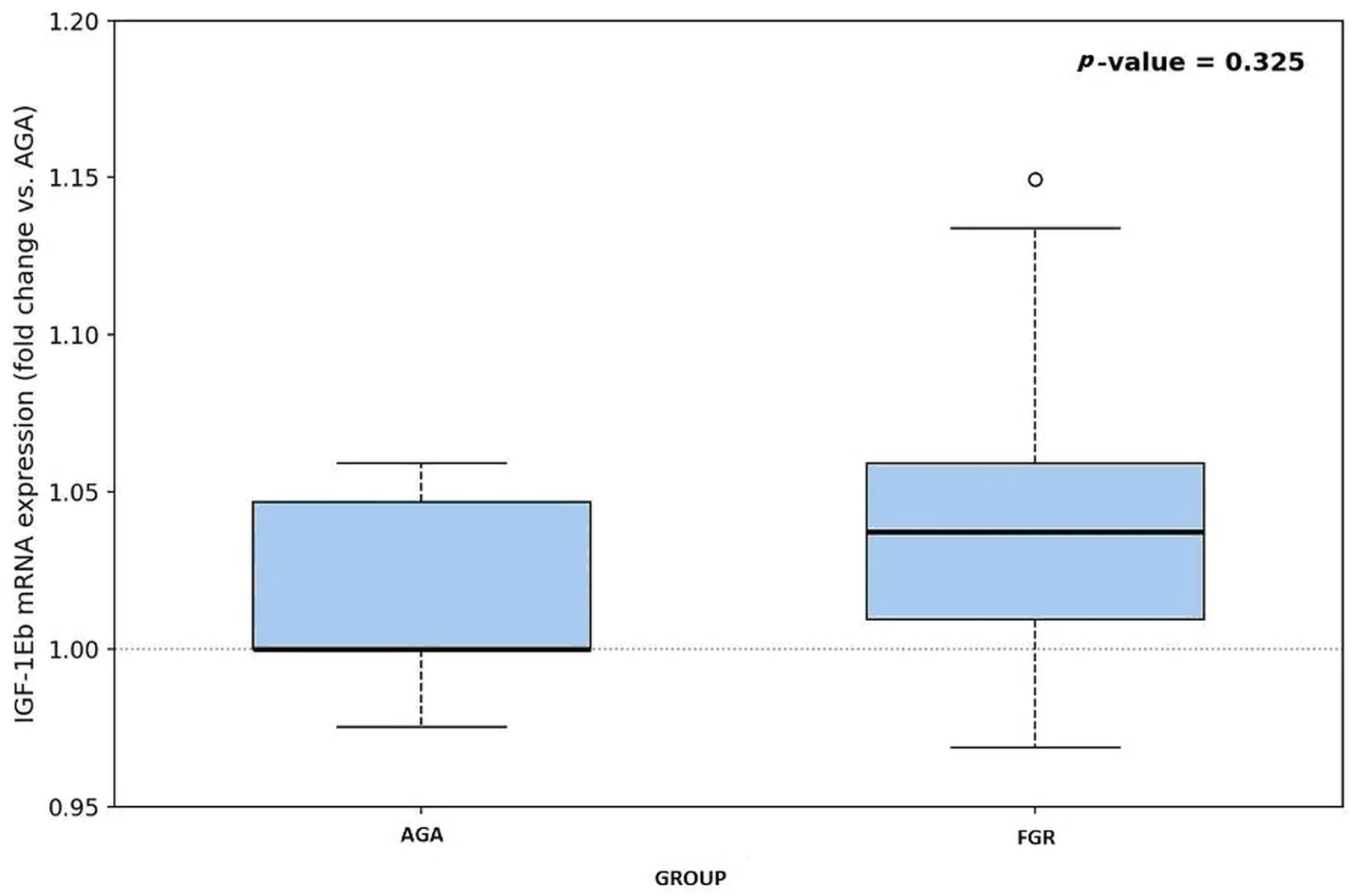
Placental IGF-1Eb mRNA expression in pregnancies complicated by FGR (n = 15) and AGA (n = 13), expressed as fold change relative to the AGA (control) group. Small circles indicate outlier values (AGA, appropriate for gestational age; IGF-1Eb, insulin-like growth factor-1Eb; FGR, fetal growth restriction).

Placental IGF-1Eb transcript abundance was quantified by qPCR, with no statistically significant difference observed between the FGR and AGA groups (*p* = 0.325; Figure 1). This null result should be interpreted in light of the precision the analysis actually affords. No a priori sample size calculation was performed. Taking the observed variability of the normalized expression values (pooled SD of ΔCt 3.03 cycles), the smallest difference detectable with 80% power was approximately 3.3 cycles, corresponding to a ten-fold change in transcript abundance. The comparison therefore provides no evidence of a difference in IGF-1Eb transcript abundance between FGR and AGA placentas, but it does not provide evidence that transcript abundance is unchanged, and it is uninformative with respect to differences in the magnitude that would be biologically plausible, i.e., biologically plausible differences between the groups cannot be excluded.

Placental IGF-1Eb mRNA levels were not significantly associated with maternal age, BMI, gestational age at delivery, fetal sex, neonatal birth weight, or placental weight. Similarly, transcript abundance did not differ significantly according to the presence of MVM, MPFD, FVM, VUE, DVM, or other histopathological placental abnormalities. Among pregnancies delivered at term (≥37 weeks of gestation), IGF-1Eb mRNA expression also remained comparable between the FGR and AGA groups. A comparable pattern was observed in pregnancies with a neonatal birth weight above 2500 g, with no significant difference in IGF-1Eb mRNA expression between FGR and AGA placentas. Likewise, in cases where placental weight exceeded 400 g, transcript levels did not differ significantly between the two groups. Each of these subgroup comparisons is based on a fraction of the 28 placentas available for mRNA analysis and is correspondingly imprecise; none of them should be read as demonstrating the absence of an association.

### 3.3. Immunostaining Localization and Distribution of IGF-1Eb in Placental Tissue

#### 3.3.1. IGF-1Eb Isoform Immunoreactivity in the Perivillous Syncytiotrophoblast of Human Placental Tissue

**Figure 2:**
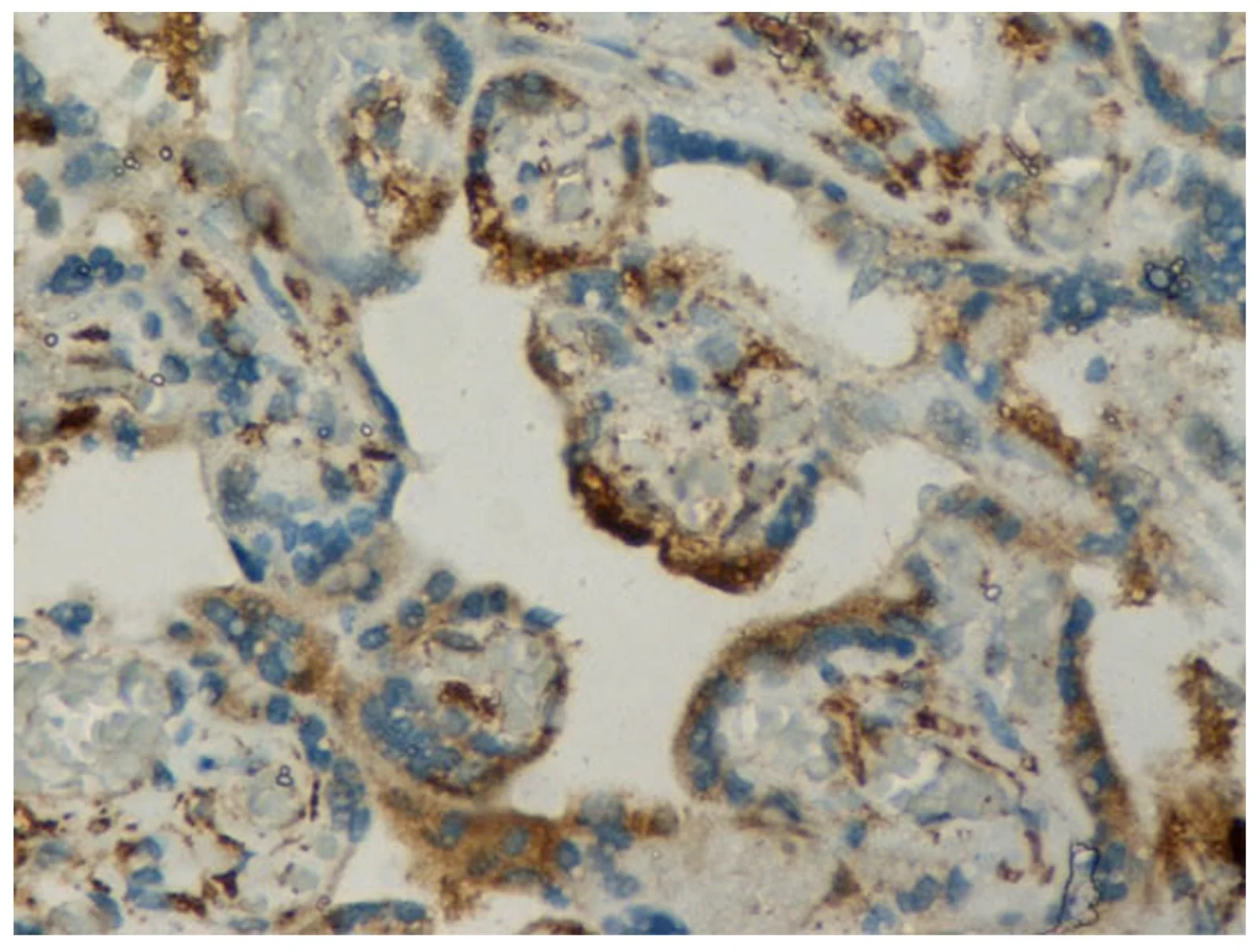
Enhanced IGF-1Eb immunoreactivity in the perivillous trophoblast at a magnification of ×200.

Immunohistochemical analysis demonstrated positive IGF-1Eb immunoreactivity in the syncytiotrophoblast, where staining appeared as granular or diffuse brown coloration localized to both the cytoplasm and the cytoplasmic membrane of perivillous cells. Representative IGF-1Eb immunopositive staining in the perivillous syncytiotrophoblast is illustrated in Figure 2.

**Figure 3:**
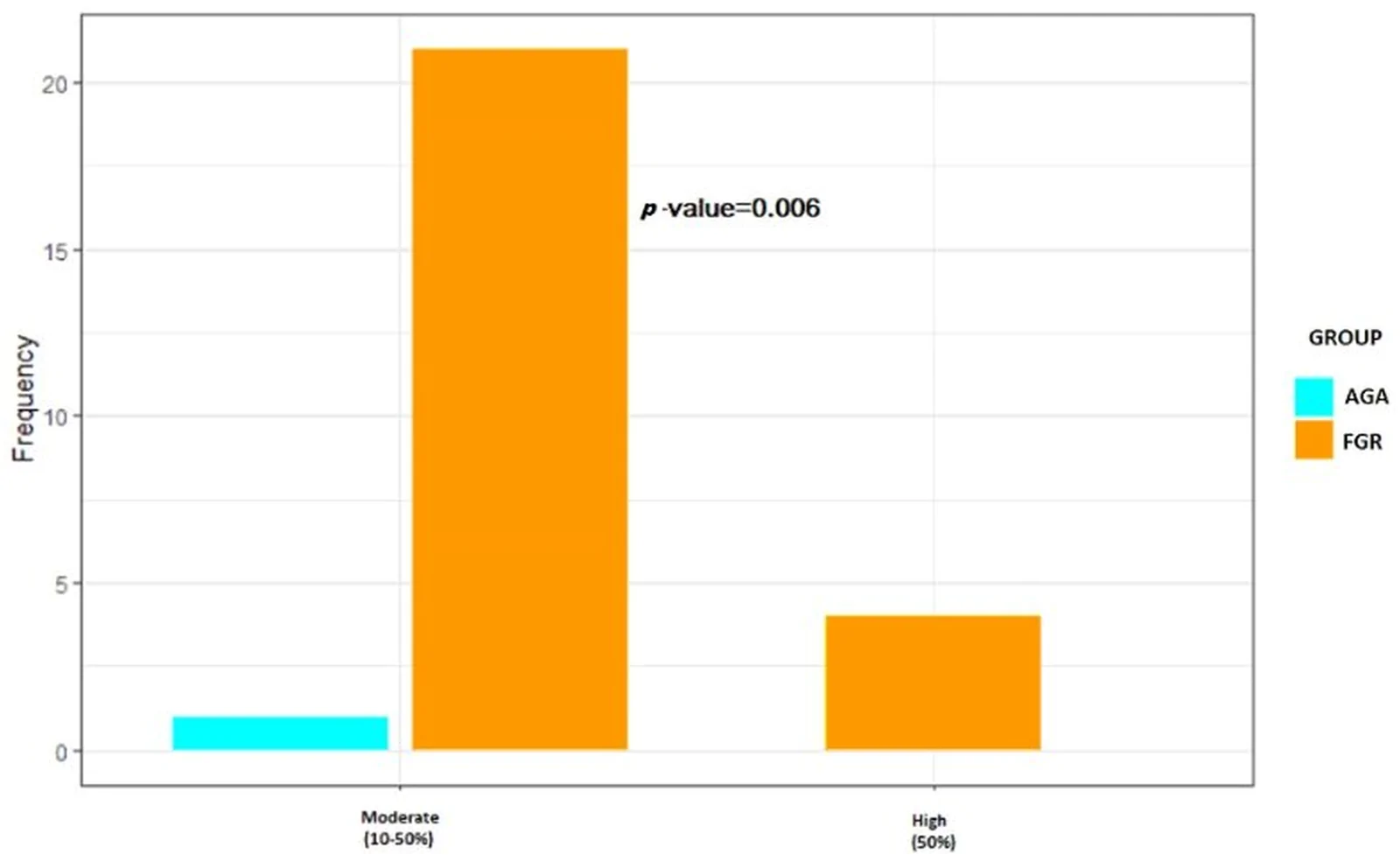
IGF-1Eb immunoreactivity scores ranging from moderate to high in perivillous syncytiotrophoblasts of third-trimester human placentas.

In a series of 47 pregnancies complicated by FGR, high IGF-1Eb immunoreactivity scores in the perivillous syncytiotrophoblast were observed in 4 cases (8.5%), while moderate immunoreactivity scores weredetected in 21 cases (44.7%). The remaining cases showed low or weak immunoreactivity scores. The remaining 22 cases (46.8%) showed no detectable immunoreactivity. By comparison, among the 15 AGA pregnancies, a moderate immunoreactivity score was observed in only 1 case (6.7%), while 14 cases (93.3%) were negative for IGF-1Eb immunoreactivity in the same placental region. A statistically significant difference in IGF-1Eb immunoreactivity patterns was found between the FGR and AGA groups (*p* = 0.006), with placentas from FGR pregnancies demonstrating a greater prevalence of moderate IGF-1Eb immunoreactivity scores within the perivillous syncytiotrophoblast than AGA placentas (Figure 3, Table 2). In particular, placentas from FGR pregnancies demonstrated a greater prevalence of moderate IGF-1Eb immunoreactivity scores within the perivillous syncytiotrophoblast than normal placentas, indicating a possible association between IGF-1Eb immunoreactivity within this compartment and the pathological processes underlying FGR (Figure 3, Table 2). IGF-1Eb immunopositivity was observed in 25 of 47 FGR placentas (53.2%) compared with 1 of 15 AGA placentas (6.7%). The corresponding effect sizes were an odds ratio of 15.34 (95% CI, 2.02–698.94), an absolute risk difference of 46.5% (95% CI, 19.5–61.1%), and a Cramér’s V of 0.40. The width of the odds-ratio interval reflects the single positive control placenta and the small size of the control group, and the risk difference is the more stable summary of the contrast.

Ιmmunoreactivity scores of IGF-1Eb in the perivillous syncytiotrophoblast differed significantly between FGR and AGA pregnancies among mothers younger than 40 years. Specifically, moderate IGF-1Eb immunoreactivity scores occurred more frequently in placental specimens from FGR pregnancies than in those from AGA pregnancies (Table 2). A significant association was also identified between IGF-1Eb immunoreactivity scores and gestational age. In pregnancies delivered at ≥37 weeks of gestation, moderate immunoreactivity scores of IGF-1Eb in the perivillous syncytiotrophoblast were more common in FGR placentas than in AGA placentas. Similarly, significant differences were found according to neonatal birth weight. Among neonates weighing ≥ 2500 g, placentas from the FGR group more frequently demonstrated moderate IGF-1Eb immunoreactivity scores in the perivillous syncytiotrophoblast compared with placentas from the AGA group (Table 2). Placental weight was likewise associated with differences in IGF-1Eb immunohistochemical scores. FGR placentas weighing ≥ 400 g showed moderate IGF-1Eb immunoreactivity scores in the perivillous syncytiotrophoblast more often than AGA placentas of similar weight (Table 2). In addition, maternal BMI < 30 kg/m^2^ was significantly related to IGF-1Eb immunoreactivity patterns. Moderate immunoreactivity scores of the IGF-1Eb isoform were identified in 63.6% of FGR cases with maternal BMI < 30 kg/m^2^, compared with only 8.3% of AGA placentas (Table 1). Fetal sex also demonstrated a significant association with IGF-1Eb immunoreactivity. Most FGR pregnancies carrying female fetuses exhibited moderate or high immunoreactivity scores of IGF-1Eb in the perivillous syncytiotrophoblast, whereas such immunoreactivity was less common in AGA placentas (Table 2). Regarding histopathological lesions, significant differences in IGF-1Eb immunoreactivity were observed between FGR and AGA placentas in cases associated with maternal vascular malperfusion (MVM) of the placental bed. Moderate immunoreactivity scores of the IGF-1Eb isoform were more strongly associated with FGR placentas than with AGA placentas in the presence of MVM (Table 2). Collectively, these findings support a potential association between increased IGF-1Eb immunoreactivity scores in the perivillous syncytiotrophoblast and the FGR placentas. These subgroup analyses were not pre-specified and are exploratory. Across the 77 estimable subgroup comparisons reported in Table 2, Table 3, Table 4, Table 5 and Table 6, 31 reached *p* < 0.05, against 3.9 significant results expected by chance under the global null hypothesis; after Benjamini–Hochberg correction at a false discovery rate of 5%, 28 remained significant. The principal findings in the perivillous syncytiotrophoblast, maternal decidual vessel endothelium, and the fetal stem and intermediate villous vessel endothelium survive correction, whereas several of the borderline associations do not and should be regarded as unconfirmed. Several strata are additionally based on very small numbers: 6 FGR and 1 AGA placenta for maternal age ≥ 40 years, 4 placentas with MPFD, 1 with FVM, 6 with VUE, 7 with DVM and 4 with other lesions. In these strata, and in those in which one arm contained no placentas and the comparison is therefore not estimable, the tabulated results are reported for completeness only; they carry no independent evidential weight and no conclusion in this manuscript rests upon them.

**Figure 4:**
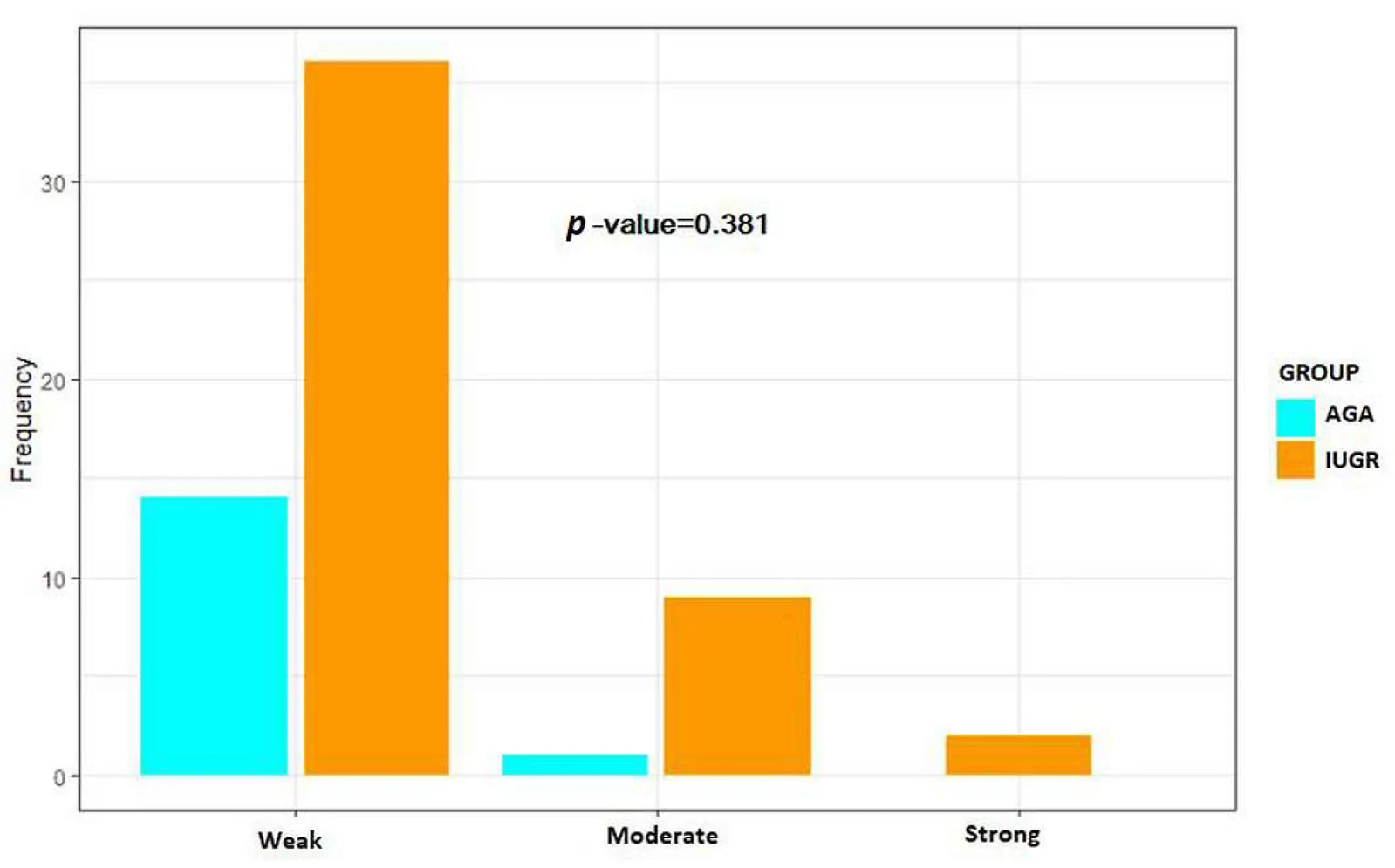
Representative weak, moderate, and strong IGF-1Eb immunoreactivity intensity in perivillous syncytiotrophoblasts of human third-trimester placentas.

Within the FGR group, strong IGF-1Eb staining intensity was identified in 2 cases (4.3%), moderate intensity in 9 cases (19.1%), and weak intensity in 36 cases (76.6%). In the AGA group, no placentas demonstrated strong staining intensity, while moderate intensity was observed in 1 case (6.7%) and weak intensity in 14 cases (93.3%). Analysis of the FGR and AGA groups showed no significant difference in the intensity of IGF-1Eb immunostaining (*p* = 0.381) (Figure 4; Table 3). With respect to histopathological findings, IGF-1Eb immunoreactivity intensity differed significantly between FGR and AGA placentas in the presence of maternal vascular malperfusion (MVM) of the placental bed (Table 3). Dichotomized as moderate or strong versus weak intensity, staining intensity was recorded in 11 of 47 FGR placentas (23.4%) and 1 of 15 AGA placentas (6.7%): odds ratio 4.20 (95% CI 0.52 to 197.07), absolute risk difference 16.7% (95% CI −8.4% to 31.6%). The confidence intervals include the null, and the data are compatible with anything from a modest reduction to a substantial increase in staining intensity.

#### 3.3.2. GF-1Eb Isoform Immunoreactivity in Placental Extravillous Trophoblasts

**Figure 5:**
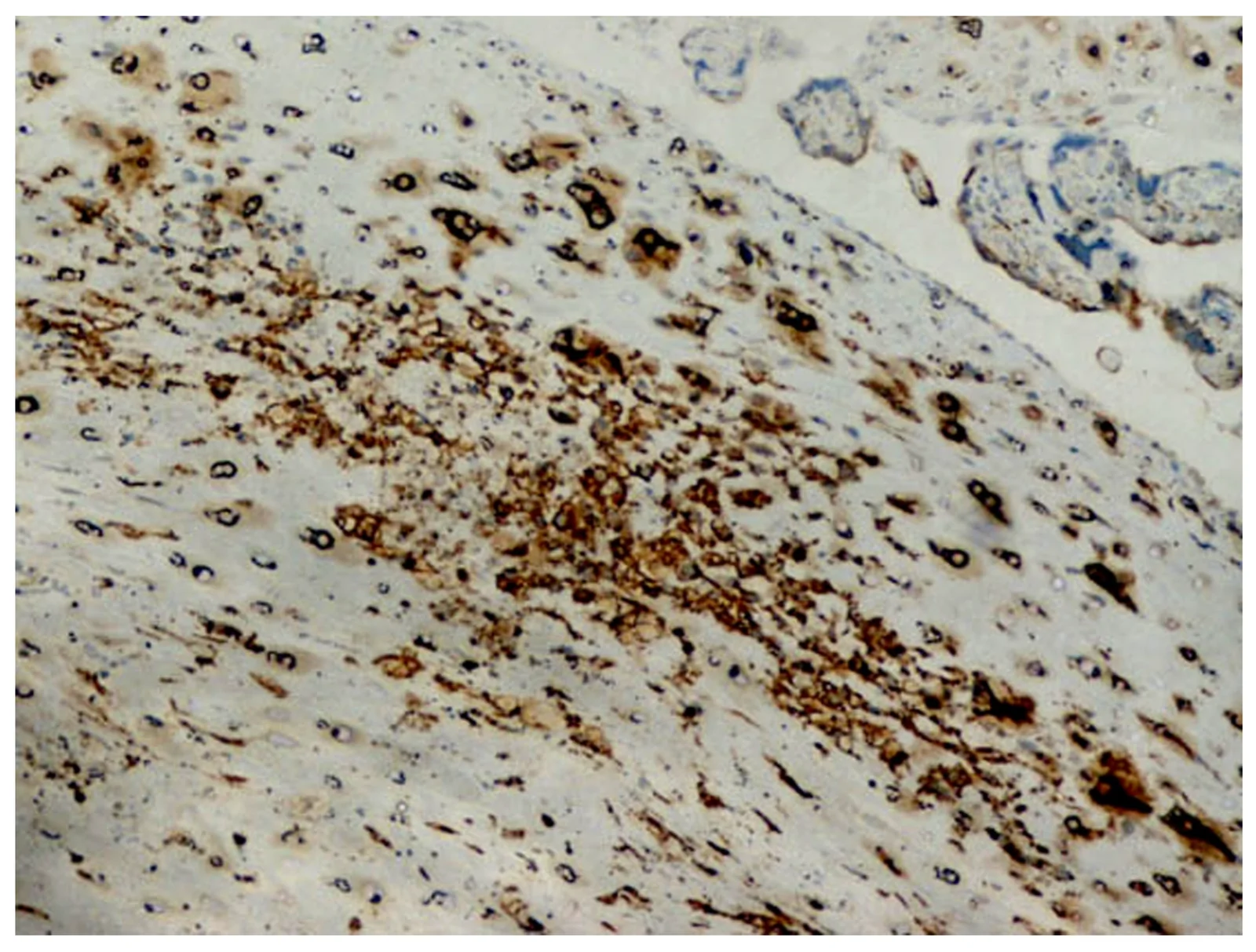
IGF-1Eb immunoreactivity in extravillous trophoblastic cells at a magnification of ×50.

IGF-1Eb immunoreactivity was detected in extravillous trophoblast cells within the basal plate and anchoring columns. Representative immunohistochemical staining demonstrating cytoplasmic and membranous IGF-1Eb localization in extravillous trophoblast is seen in Figure 5.

**Figure 6:**
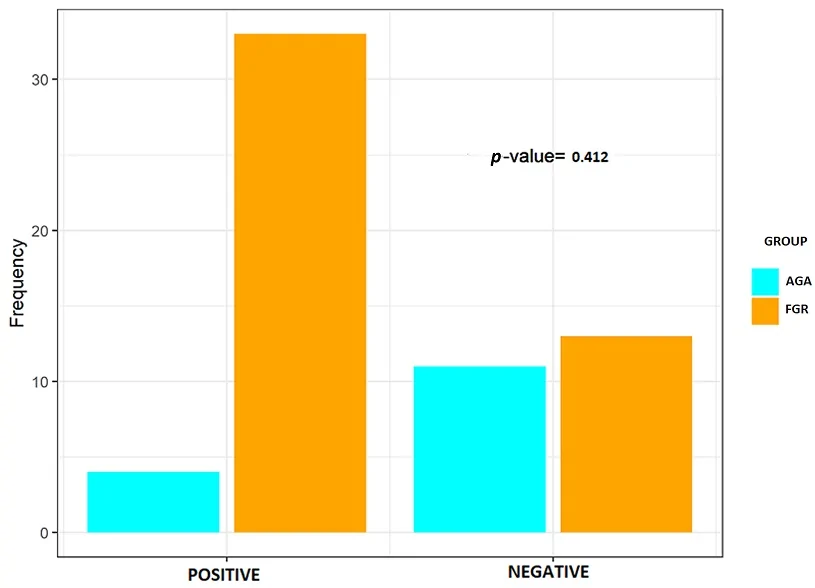
Positive and negative IGF-1Eb immunoreactivity in extravillous cytotrophoblasts of human placentas obtained during third-trimester pregnancies.

Immunohistochemical positivity and staining intensity for IGF-1Eb in extravillous trophoblastic cells did not differ significantly between FGR and AGA placentas (*p* = 0.412). Positive IGF-1Eb staining was detected in 73.3% of AGA placentas and in 61.7% of FGR placentas (Figure 6; Table 4). Similarly, there were no significant associations of IGF-1Eb immunoreactivity in the extravillous trophoblast with histopathological findings or with clinical characteristics, including maternal age, neonatal birth weight exceeding 2500 g, placental weight above 400 g, maternal BMI, and fetal sex (Table 4). The corresponding effect size was an odds ratio of 0.59 (95% CI 0.12 to 2.40) with an absolute risk difference of −11.6% (95% CI −32.9% to 16.6%), and Cramér’s V was 0.10. The confidence interval is wide and does not exclude a moderate difference in either direction; the appropriate conclusion is that no difference was detected rather than that extravillous trophoblast immunoreactivity is equivalent between groups.

**Figure 7:**
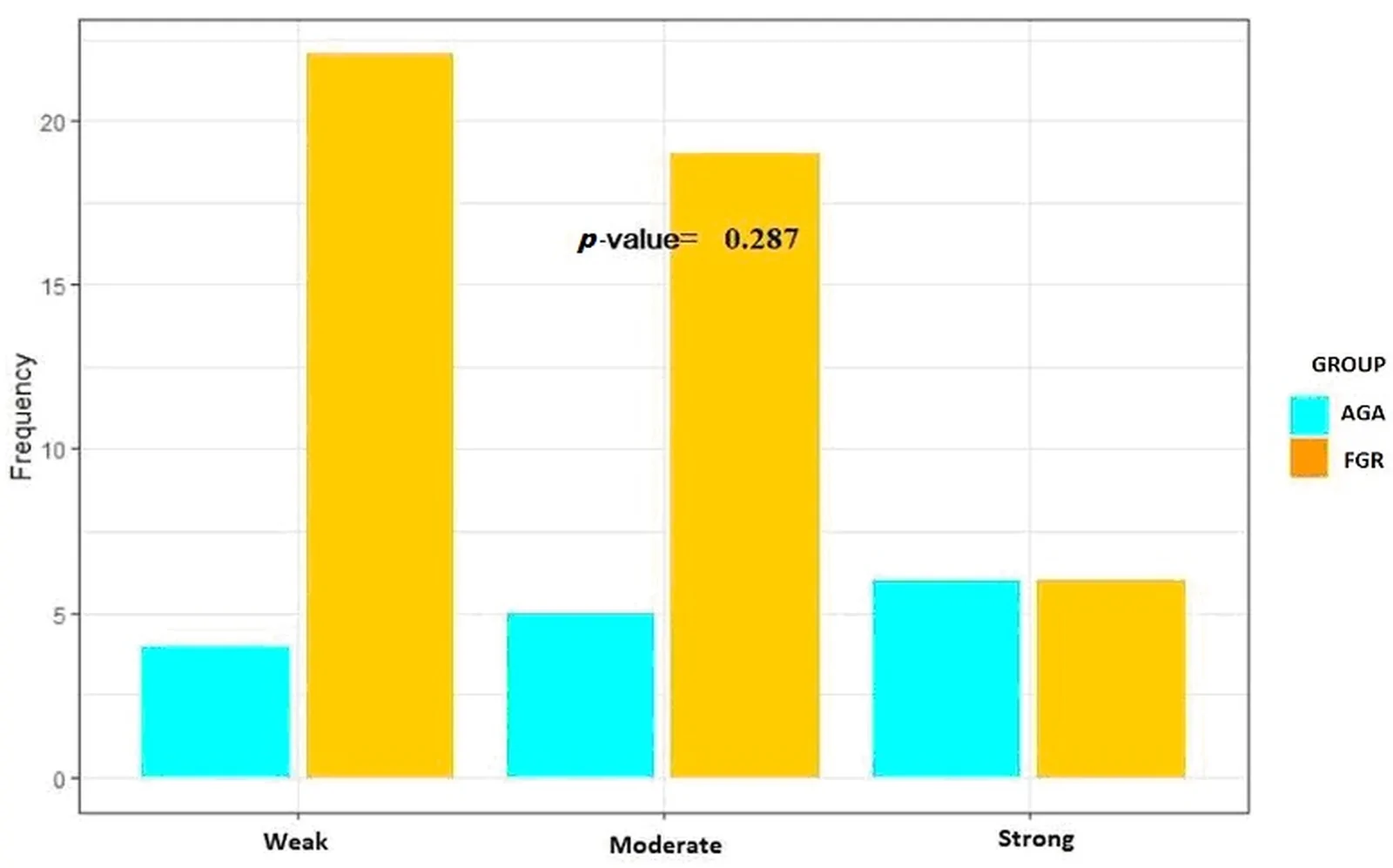
Intensity of IGF-1Eb immunoreactivity.

No significant difference in IGF-1Eb immunostaining intensity within extravillous trophoblast cells was observed between FGR and AGA pregnancies (Figure 7; Table 5). Moreover, IGF-1Eb immunostaining intensity was not significantly associated with maternal age, neonatal birth weight above 2500 g, placental weight over 400 g, maternal BMI, fetal sex, or the presence of maternal vascular malperfusion (MVM).

#### 3.3.3. IGF-1Eb Immunoreactivity in Maternal Decidual and Fetal Villous Vascular Endothelium of Human Placentas

**Figure 8:**
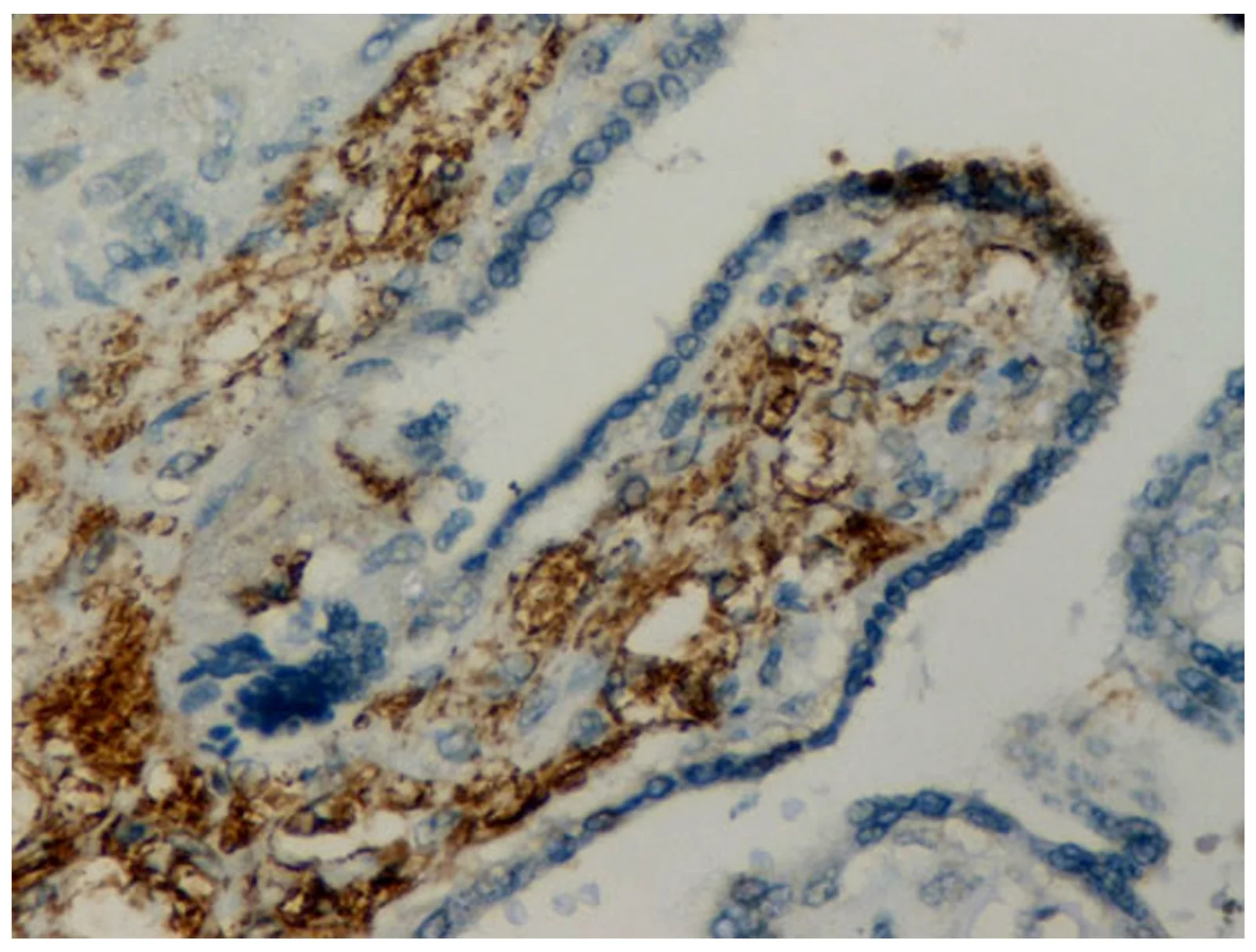
IGF-1Eb immunoreactivity in the endothelium of fetal villous vessels in an FGR placenta at a magnification of ×200. Positive perivillous trophoblastic cells are also seen on the tip of the chorionic villous.

IGF-1Eb immunoreactivity was additionally identified in the endothelial lining of fetal vessels throughout the stem, intermediate, and distal chorionic villi, as well as in maternal decidual vessels. Figure 8 illustrates IGF-1Eb immunostaining in fetal villous vessels from an FGR placenta.

A significant difference in IGF-1Eb immunopositivity of the maternal decidual vascular endothelium was observed between FGR and AGA placentas from third-trimester pregnancies (*p* < 0.001) (Table 6). Likewise, IGF-1Eb immunopositivity in the endothelial cells of fetal vessels located in the stem and intermediate villi was significantly higher in FGR placentas than in AGA placentas (*p* < 0.001) (Table 6). Conversely, IGF-1Eb immunoreactivity in the endothelium of fetal vessels within the distal peripheral villi did not differ significantly between the FGR and AGA groups (*p* = 0.061). The corresponding effect sizes were substantial (Table 7). For the endothelium of maternal decidual vessels, IGF-1Eb was positive in 35 of 47 FGR placentas (74.5%) versus 2 of 15 AGA placentas (13.3%): odds ratio 17.91 (95% CI 3.36 to 186.09), absolute risk difference 61.1% (95% CI 32.9% to 75.2%), Cramér’s V 0.53. For the endothelium of fetal stem and intermediate villous vessels, positivity was recorded in 36 of 47 FGR placentas (76.6%) versus 3 of 15 AGA placentas (20.0%): odds ratio 12.43 (95% CI 2.72 to 81.07), absolute risk difference 56.6% (95% CI 27.9% to 72.8%), Cramér’s V 0.50. For the endothelium of fetal distal–peripheral villous vessels the difference did not reach significance: 20 of 46 FGR placentas (43.5%) versus 2 of 15 AGA placentas (13.3%), odds ratio 4.89 (95% CI 0.94 to 49.53), absolute risk difference 30.1% (95% CI 2.2% to 47.3%).

In FGR placentas, IGF-1Eb immunoreactivity in the endothelial cells of maternal decidual vessels was significantly associated with maternal age, gestational age, neonatal birth weight, placental weight, maternal BMI, fetal sex, and the presence of maternal vascular malperfusion (MVM) lesions in the placental bed, compared with AGA placentas (Table 6). Likewise, in FGR placentas, IGF-1Eb immunopositivity within the endothelium of fetal stem and intermediate villous vessels showed significant associations with maternal age, gestational age, neonatal and placental weights, maternal BMI, fetal sex, and the presence of MVM lesions in the placental bed, in comparison with AGA placentas (Table 6). Furthermore, among FGR placentas, IGF-1Eb immunoreactivity in the endothelium of fetal distal peripheral vessels was significantly associated with gestational age, neonatal birth weight, placental weight, maternal BMI, and female fetal sex compared with AGA placentas (Table 6).

### 3.4. Multivariable Analysis

Because gestational age at delivery, maternal age, fetal sex and maternal vascular malperfusion are closely interrelated and differ systematically between the two groups, multivariable models were fitted to examine whether the association between IGF-1Eb immunoreactivity and FGR persisted after adjustment for the available covariates. All models were estimated by Firth’s penalized likelihood, since several strata were completely separated and ordinary maximum likelihood is undefined in that situation. Neonatal birth weight and placental weight were not included as covariates because they are closely related characteristics of the FGR phenotype rather than variables antecedent to it, and are therefore unsuitable as conventional confounders in this analysis. Preeclampsia status could not be established for every case and could not be entered into any model. The results are summarized in Table 8.

After adjustment for gestational age at delivery (Model 1, all 62 placentas), the association between FGR and IGF-1Eb positivity persisted in the perivillous syncytiotrophoblast (adjusted odds ratio 7.36, 95% CI 1.38 to 76.01; *p* = 0.017), in the endothelium of maternal decidual vessels (adjusted odds ratio 14.87, 95% CI 3.19 to 96.63; *p* < 0.001) and in the endothelium of fetal stem and intermediate villous vessels (adjusted odds ratio 10.68, 95% CI 2.47 to 56.99; *p* = 0.001). Positivity in the endothelium of fetal distal–peripheral villous vessels, which did not reach significance in the unadjusted comparison (*p* = 0.061), reached significance once gestational age was accounted for (adjusted odds ratio 5.73, 95% CI 1.27 to 35.63; *p* = 0.022). Gestational age itself was not significantly associated with any of the endothelial endpoints in these models. Neither extravillous trophoblast positivity (adjusted odds ratio 0.36, 95% CI 0.08 to 1.43; *p* = 0.149) nor extravillous staining intensity (adjusted odds ratio 0.32, 95% CI 0.07 to 1.23; *p* = 0.099) was associated with FGR, and neither was perivillous staining intensity (adjusted odds ratio 1.29, 95% CI 0.20 to 14.42; *p* = 0.800).

These adjusted estimates should not be taken to indicate that gestational-age-related confounding has been removed. Because all AGA placentas were delivered at term and appropriately grown preterm controls were unavailable, the two groups do not overlap below 37 weeks of gestation, and adjustment for gestational age cannot substitute for gestational-age-matched comparison. Gestational age at delivery is moreover influenced in FGR pregnancies by disease severity and by the timing of clinical intervention, so it does not behave as a conventional baseline confounder. Differences observed in preterm FGR placentas may therefore partly reflect placental maturation rather than growth restriction itself, and this possibility applies to the adjusted models as much as to the unadjusted comparisons.

Model 2, which additionally adjusted for maternal age and fetal sex in the 40 placentas with complete covariate data, was conducted as a sensitivity analysis. The direction and persistence of every association were maintained, and fetal sex was not associated with any endpoint once study group was included in the model. The adjusted odds ratios obtained in this model are, however, very large and are accompanied by extremely wide confidence intervals, because the data remain sparse and fewer than two thirds of the cohort contribute. They should be interpreted as indicating only that the associations do not disappear when these covariates are added; their numerical magnitude should not be interpreted as an effect estimate. The corresponding values are reported in Table 8 for completeness.

Adjustment for maternal vascular malperfusion (Model 3) left the group effect essentially unchanged: adjusted odds ratios of 7.65 (95% CI 1.43 to 79.23; *p* = 0.015) for the perivillous syncytiotrophoblast, 14.19 (95% CI 3.07 to 91.14; *p* < 0.001) for maternal decidual vessels, 10.24 (95% CI 2.40 to 53.79; *p* = 0.001) for fetal stem and intermediate vessels and 6.24 (95% CI 1.35 to 39.95; *p* = 0.018) for fetal distal–peripheral vessels. Maternal vascular malperfusion was not itself significantly associated with IGF-1Eb positivity in any compartment once study group was included in the model (for example, adjusted odds ratio 2.01, 95% CI 0.57 to 7.73, *p* = 0.280 for the perivillous syncytiotrophoblast, and adjusted odds ratio 0.77, 95% CI 0.18 to 2.88, *p* = 0.699 for maternal decidual vessels). This suggests that the association between IGF-1Eb immunoreactivity and maternal vascular malperfusion described above is largely attributable to the higher frequency of this lesion in growth-restricted placentas rather than to a lesion-specific effect, and the interpretation offered in the Discussion has been qualified accordingly.

Taken together, these analyses indicate that the compartment-specific increases in IGF-1Eb immunoreactivity are not accounted for by maternal age, fetal sex or maternal vascular malperfusion, and that they persist after adjustment for the available covariates. They do not establish that the pattern is specific to FGR. Preeclampsia status could not be ascertained for every case and could not be modeled, and the present design cannot distinguish an alteration specific to FGR from one associated with preeclampsia-related placental dysfunction, or from a placental phenotype common to both. Confounding by gestational age and placental maturation is reduced but not removed, since no preterm stratum contains an AGA placenta. Residual confounding, in particular by the indication for delivery and by variables that were not recorded, cannot be excluded, and the confidence intervals remain wide throughout.

Model 1 adjusts for gestational age at delivery; Model 2 additionally adjusts for maternal age and fetal sex and is restricted to the 40 placentas with complete covariate data; Model 3 adjusts for gestational age and maternal vascular malperfusion. Model 2 is a sensitivity analysis: its adjusted odds ratios are large and imprecise because the data are sparse, and they indicate persistence of the direction of association rather than providing quantitative effect estimates. Neonatal birth weight and placental weight were not adjusted for because they are closely related characteristics of the FGR phenotype rather than variables antecedent to it. Preeclampsia status could not be established for every case and is not included in any model, so all estimates reflect adjustment for the available covariates only. Adjustment for gestational age reduces but does not eliminate confounding by gestational age and placental maturation and does not substitute for gestational-age-matched controls. Confidence intervals are profile penalized-likelihood intervals and *p*-values are penalized likelihood-ratio tests.

## 4. Discussion

The phosphatidylinositol-3-kinase (PI3K)/Akt pathway is a major downstream signaling cascade of the insulin-like growth factor-1 receptor (IGF-1R), contributing substantially to placental growth and development, trophoblast differentiation, vascular formation, and the control of nutrient delivery to the fetus [20,21]. Accumulating evidence suggests that disruption of PI3K/Akt signaling may contribute to placental insufficiency and the development of FGR, which is primarily associated with compromised placental function and restricted fetal growth [22]. Fetal growth restriction (FGR) was defined by the Society for Maternal-Fetal Medicine (SMFM) as a sonographic estimated fetal weight (EFW) below the 10th percentile for gestational age [23]. Appropriate-for-gestational-age (AGA) pregnancies were defined as uncomplicated pregnancies with an estimated fetal weight (EFW) between the 10th and 90th percentiles for gestational age. For inclusion in the present study, a more stringent EFW threshold of greater than fifth percentile was applied to the FGR group in order to enrich the cohort for more severe forms of growth restriction; this threshold was therefore used as a study-specific inclusion criterion rather than as an alternative diagnostic definition of FGR. Despite substantial investigation of the broader IGF signaling axis in growth-restricted pregnancies, the expression and immunoreactivity profile and biological significance of individual IGF-1 splice variants in placental pathology remain insufficiently understood. To the best of our knowledge, this is the first study to examine IGF-1Eb isoform mRNA expression and immunoreactivity patterns in human placental tissue from singleton pregnancies characterized by either normal fetal growth or FGR. Despite the potential relevance of this isoform to placental function and fetal growth, its physiological expression across gestation in an AGA population has not yet been characterized. Consequently, the normal trajectory of placental IGF-1Eb mRNA expression and immunoreactivity patterns throughout pregnancy remains unclear. The present study provides preliminary data on IGF-1Eb mRNA expression and immunoreactivity patterns in placental tissue collected during the third trimester; however, the cross-sectional nature of the study does not allow conclusions to be drawn regarding changes in expression and immunopositivity according to gestational age. Longitudinal or cross-sectional studies including AGA pregnancies at multiple gestational stages are therefore needed to define the physiological pattern of IGF-1Eb expression and immunopositivity and to determine whether deviations from this trajectory are related to placental dysfunction or impaired fetal growth.

The reliability of the molecular findings was supported by rigorous quality control of the RNA samples used for mRNA expression analysis. RNA concentration and purity were evaluated spectrophotometrically, with 260/280 ratios within the expected range of 1.8–2.0, while integrity was further confirmed by electrophoretic assessment of the 18S and 28S ribosomal RNA bands. In addition, 18S rRNA was employed as an endogenous reference to normalize for potential differences in the amount of input material and reverse-transcription efficiency. Collectively, these procedures ensured adequate RNA quality and provided a robust basis for the subsequent analysis of IGF-1Eb mRNA expression.

An additional strength of the study is the combined assessment of IGF-1Eb at both the transcriptional and tissue levels, together with its relationship to clinical and histopathological characteristics. These findings should be interpreted as complementary to, rather than directly comparable with, previous genetic investigations, including genome-wide association studies (GWASs) and other genetic association analyses. Whereas such studies focus primarily on inherited genetic variation and its statistical relationship with fetal growth, placental function, or pregnancy-related phenotypes, the present investigation addresses placental IGF-1Eb mRNA abundance and compartment-specific protein localization within the tissue. Accordingly, the current data neither demonstrate a genetic association nor establish whether differences in IGF-1Eb immunoreactivity are attributable to specific genetic variants. Instead, they provide tissue-level evidence suggesting that altered placental IGF-1Eb immunoreactivity may be associated with distinct histopathological characteristics observed in FGR.

Future studies integrating placental IGF-1Eb expression and protein localization with genetic information from GWASs and other well-characterized association studies could help determine whether inherited genetic variation contributes to differences in IGF-1Eb regulation. Such an integrated approach may also provide further insight into the molecular mechanisms linking placental IGF-1Eb dysregulation with placental pathology and impaired fetal growth. The principal findings of this investigation can be summarized as follows. First, no significant difference in placental IGF-1Eb mRNA levels was detected between FGR and AGA pregnancies. Second, immunohistochemical analysis demonstrated significantly increased IGF-1Eb protein localization within the perivillous syncytiotrophoblast and the vascular endothelium of maternal decidual and fetal villous vessels in placentas complicated by FGR. Third, extravillous trophoblastic immunoreactivity remained largely unchanged between groups. Finally, enhanced endothelial immunoreactivity exhibited strong associations with histopathological evidence of maternal vascular malperfusion (MVM). Collectively, these findings suggest that placental IGF-1Eb regulation in FGR is compartment-specific and predominantly governed by post-transcriptional mechanisms rather than by altered gene transcription alone. It is important to note that the present study used the same previously characterized placental cohort [13], which included some pregnancies associated with preeclampsia. Therefore, the observed differences in IGF-1Eb immunoreactivity cannot be attributed specifically to idiopathic FGR, and the potential contribution of preeclampsia should be considered when interpreting the findings. This limitation precludes conclusions regarding IGF-1Eb alterations that are specific to idiopathic FGR and underscores the need for future prospective studies using well-characterized cohorts with predefined exclusion criteria.

The biological actions of IGF-1 are mediated predominantly through its interaction with the IGF-1 receptor (IGF-1R), a transmembrane tyrosine kinase receptor abundantly expressed in placental trophoblasts and endothelial cells [24,25]. Upon ligand engagement, IGF-1R undergoes autophosphorylation and interacts with insulin receptor substrate (IRS) proteins, triggering downstream signaling networks, particularly the PI3K/Akt and mitogen-activated protein kinase (MAPK) pathways [26]. Among these pathways, PI3K/Akt signaling appears particularly relevant to placental biology because it regulates trophoblast proliferation, resistance to apoptosis, glucose uptake, protein synthesis, angiogenesis, and nutrient transporter activity [25,27]. Experimental evidence indicates that Akt activation promotes trophoblast survival and differentiation while simultaneously enhancing placental nutrient transport through modulation of amino acid and glucose transporter expression [28].

Impairment of this signaling network has been consistently reported in growth-restricted pregnancies. Previous studies have demonstrated reduced placental IGF signaling activity, including diminished IGF-1R phosphorylation and attenuated downstream Akt activation in FGR placentas [19,20,21,22,23,24,25,26,27,28,29,30,31]. Such disruption may contribute directly to trophoblastic dysfunction by limiting proliferative capacity and increasing susceptibility to oxidative and hypoxic injury, pathological conditions frequently observed in placental insufficiency [32,33,34]. Reduced PI3K/Akt activity may additionally compromise mTOR signaling and thereby impair nutrient transport and fetal growth [35,36]. Within this biological framework, preservation or selective upregulation of specific IGF-related proteins may constitute a compensatory mechanism aimed at maintaining placental homeostasis despite impaired upstream signaling [37].

The apparent discrepancy between unchanged IGF-1Eb mRNA expression and significantly increased protein immunoreactivity in FGR placentas strongly supports this interpretation. Divergence between transcript abundance and protein-level immunoreactivity is increasingly recognized in placental biology and reflects the complex regulatory landscape governing trophoblast adaptation [38,39]. Protein abundance and detectability are influenced not only by transcription but also by translational efficiency, post-transcriptional modification, intracellular trafficking, degradation kinetics, and local tissue retention. These mechanisms become particularly relevant under conditions of placental stress, where adaptive responses frequently occur at translational and post-translational levels rather than through changes in gene expression alone [40]. The apparent discrepancy between unchanged IGF-1Ea mRNA expression and increased immunohistochemical protein positivity may reflect both biological regulation at the post-transcriptional or translational level and methodological differences inherent to the assessment of transcripts and protein. Unchanged overall transcript abundance does not necessarily imply unchanged protein production, as differences in translational efficiency, protein stability, or degradation rates may result in altered protein accumulation without a corresponding change in mRNA levels. In addition, changes in the structural or activation state of the protein may affect its immunohistochemical detectability without necessarily reflecting a proportional change in its absolute abundance. This possibility is consistent with the concept that mRNA and protein levels are not invariably coupled and may be subject to distinct regulatory mechanisms [41]. At the same time, the intrinsic histological heterogeneity of the placenta may have contributed to the observed discrepancy. IGF-1Eb immunoreactivity varied among different placental structures, including the endothelium of maternal decidual and fetal villous vessels and the perivillous syncytiotrophoblast, and was therefore assessed across multiple optical fields using a semi-quantitative approach. In contrast, mRNA expression was determined from a focal frozen placental sample, providing an overall measure of transcript abundance within that particular tissue specimen and integrating the contribution of different cellular and histological components (13). Consequently, differences in tissue composition and sampling, together with the possibility of differential post-transcriptional, translational, or protein-stability regulation, may account for the apparent discordance between IGF-1Eb transcript and protein immunopositivity in FGRand AGA placentas.

FGR is especially relevant in the context of hypoxia, a hallmark of placental insufficiency and maternal vascular malperfusion. Oxygen availability represents a key regulatory factor in placental development and the differentiation of trophoblast cells [42]. Physiological hypoxia during early gestation supports normal placental morphogenesis and trophoblast invasion; however, persistent or pathological hypoxia later in pregnancy activates stress-response pathways capable of disrupting placental structure and function [43,44]. A key component of this adaptive response is the stabilization of hypoxia-inducible factors (HIFs), especially HIF-1α, which modulate the expression of genes governing angiogenesis, cellular metabolism, proliferation, and survival [45,46,47,48]. Sustained HIF signaling has been documented in placentas complicated by fetal growth restriction and preeclampsia, supporting the concept that chronic hypoxic stress contributes substantially to placental dysfunction [49,50,51].

Under such conditions, regulation of protein synthesis may become uncoupled from transcriptional activity. Hypoxia influences translational control, peptide processing, and protein secretion through both HIF-dependent and independent mechanisms [52,53]. Consequently, the increased IGF-1Eb immunoreactivity identified in FGR placentas may reflect enhanced translation, altered protein turnover, or localized accumulation rather than increased transcriptional activation. The present findings therefore suggest that the IGF-1Eb isoform participates in adaptive stress responses activated within the growth-restricted placenta.

A particularly notable finding was the increased IGF-1Eb immunoreactivity within the perivillous syncytiotrophoblast. Moderate syncytiotrophoblastic immunoreactivity occurred significantly more frequently in FGR placentas and demonstrated meaningful associations with gestational age, placental weight, neonatal birth weight, maternal body mass index, fetal sex, and maternal vascular malperfusion. These associations indicate that regulation of this splice variant may be influenced by both maternal and fetal determinants and further support the biological relevance of IGF-1Eb in placental adaptation.

The syncytiotrophoblast constitutes the primary maternal–fetal exchange interface and serves essential transport, endocrine, and immunological functions [49,54,55,56]. Through extensive contact with maternal blood, this multinucleated epithelium regulates transfer of oxygen, glucose, amino acids, and other nutrients necessary for fetal growth. IGF-1 signaling within this compartment has been recognized as a major regulator of trophoblast proliferation, survival, and nutrient transporter expression [30,57]. Engagement of IGF-1R activates the PI3K/Akt axis and its downstream mammalian target of rapamycin (mTOR) signaling, supporting metabolic activity and the trafficking of nutrient transporters [58,59,60]. Reduced Akt and mTOR activity documented in FGR placentas correlates closely with impaired amino acid transport and reduced fetal growth [61,62].

Accordingly, increased syncytiotrophoblastic IGF-1Eb immunoreactivity may represent an adaptive attempt to compensate for defective signaling and preserve trophoblast function. Similar compensatory upregulation of growth factor pathways has been reported in placentas exposed to chronic hypoxia and nutrient deprivation [61,63,64,65]. Rather than indicating increased absolute IGF-1Eb protein abundance, enhanced IGF-1Eb immunoreactivity may reflect a protective mechanism aimed at sustaining trophoblast viability and maternal–fetal nutrient exchange in the setting of placental insufficiency.

An additional observation of particular interest was the association between syncytiotrophoblastic IGF-1Eb immunoreactivity and fetal sex. Increased immunoreactivity was more frequently identified in placentas from FGR pregnancies carrying female fetuses, suggesting that fetal sex may influence regulation of this splice variant and potentially modulate placental adaptive responses to growth restriction. Increasing evidence indicates that placental function exhibits substantial sexual dimorphism, involving differences in endocrine activity, immune regulation, nutrient handling, and stress-response pathways [66,67]. These sex-specific differences may be particularly important in complicated pregnancies, as male and female placentas may employ distinct adaptive responses to unfavorable intrauterine environments [68]. Placental gene expression profiles have been shown to differ significantly according to fetal sex in growth-restricted pregnancies, with female and male placentas exhibiting distinct molecular signatures and pathway enrichment patterns [69,70]. Female placentas have frequently been proposed to adopt a more adaptive or resilient phenotype, prioritizing maintenance of placental function and fetal survival under conditions of environmental stress, whereas male placentas may exhibit comparatively limited compensatory flexibility. Although the biological basis of these sex-dependent differences has not yet been fully elucidated, they may arise from complex interactions involving sex hormones, epigenetic mechanisms, mitochondrial function, and immune-related signaling [66,67,68,69,70,71,72,73]. Within this context, the present findings raise the possibility that IGF-1Eb forms part of a sex-specific adaptive program activated in growth-restricted pregnancies. While this interpretation remains speculative, it highlights an important avenue for future investigation and underscores the necessity of considering fetal sex as a biologically relevant variable in placental research.

The present findings also extend previous observations regarding placental IGF expression and immunoreactivity in FGR pregnancies. Earlier immunohistochemical studies identified altered IGF localization within placental trophoblast and vascular compartments and proposed a role for IGF signaling in local adaptive responses to fetal compromise. Dalçik et al. observed elevated placental IGF immunoreactivity in FGR cases, whereas Özkan et al. subsequently reported increased IGF-I staining in trophoblastic cells from pregnancies involving small-for-gestational-age fetuses [74,75]. Studies of the wider IGF signaling system, encompassing receptor expression and downstream pathway activity, have likewise highlighted the importance of IGF-dependent mechanisms in fetal growth and placental adaptive responses [76,77,78].

However, those studies primarily evaluated total IGF-I expression and immunoreactivity and therefore could not distinguish between alternative splice variants. The current study expands this literature by demonstrating isoform-specific alterations involving IGF-1Eb. Such findings are biologically important because alternative splicing of the IGF-1 gene generates distinct E-peptides that may possess functions extending beyond those of mature IGF-1 itself [17]. Although the physiological role of IGF-1Eb in placental tissue remains incompletely characterized, evidence from skeletal muscle biology and regenerative medicine suggests that IGF-1Eb-derived peptides participate in tissue remodeling, cellular proliferation, and stress adaptation [79,80]. These observations support the possibility that individual IGF-1 splice variants may serve specialized and nonredundant functions within the placenta.

This concept is reinforced by the compartment-specific localization patterns identified in the present study. In contrast to the marked increase observed within syncytiotrophoblastic and endothelial compartments, extravillous trophoblast (EVT) immunopositivity of IGF-1Eb remained stable across study groups. Extravillous trophoblast (EVT) cells are essential for remodeling the maternal spiral arteries and establishing sufficient uteroplacental blood flow during early pregnancy [81,82]. Because these processes are largely completed by mid-pregnancy, the absence of significant differences in third-trimester EVT immunoreactivity may indicate that IGF-1Eb has a limited role in late EVT physiology or that its activity within this compartment is temporally restricted to earlier stages of placentation.

The stability of EVT immunoreactivity may therefore reflect developmental specificity rather than biological irrelevance. The absence of significant differences in third-trimester EVT immunoreactivity does not exclude the possibility that IGF-1Eb may be relevant to trophoblast invasion or vascular remodeling during earlier stages of placentation; however, this possibility cannot be evaluated from the present late-gestation placental specimens and requires dedicated functional and longitudinal studies. Alternatively, differential regulation among trophoblast subtypes may indicate functional specialization of IGF-1 isoforms. The selective increase observed within syncytiotrophoblast and endothelial cells, together with unchanged EVT immunopositivity, supports the hypothesis that alternative IGF-1 isoforms participate in distinct and compartment-specific biological processes rather than representing interchangeable molecular products.

A notable finding was the association between IGF-1Eb immunoreactivity and maternal vascular malperfusion. MVM represents a major histopathological feature of placental insufficiency and is associated with impaired uteroplacental perfusion and chronic placental ischemia [80,81,82]. Histopathological features including distal villous hypoplasia, accelerated villous maturation, placental infarction, and decidual arteriopathy are indicative of persistent vascular dysfunction and have been strongly linked to FGR and unfavorable perinatal outcomes.

The enhanced endothelial IGF-1Eb immunoreactivity identified in placentas exhibiting MVM therefore warrants particular consideration. Placental angiogenesis and vascular homeostasis are critically regulated by IGF-1R-mediated PI3K/Akt signaling, which supports endothelial survival, nitric oxide production, migration, and vascular remodeling [83,84,85,86,87,88,89]. Akt signaling enhances endothelial nitric oxide synthase (eNOS) activity, thereby increasing nitric oxide production and promoting vasodilation and adequate uteroplacental blood flow [90,91]. Impairment of endothelial PI3K/Akt signaling has been documented in placentas complicated by FGR and preeclampsia and contributes to vascular dysfunction and abnormal fetoplacental blood flow [92,93,94]. This interpretation requires two qualifications. First, in the multivariable analysis maternal vascular malperfusion was not itself associated with IGF-1Eb immunoreactivity once study group was accounted for, indicating that the association observed in the unadjusted analysis is largely attributable to the higher frequency of this lesion in FGR placentas rather than to a lesion-specific effect. Second, because preeclampsia status could not be established for every case, the present data cannot determine whether the IGF-1Eb pattern described here is specific to FGR, reflects preeclampsia-associated placental dysfunction, or represents a placental phenotype shared by both.

The enhanced endothelial IGF-1Eb immunoreactivity observed in placentas exhibiting MVM is consistent with an association between IGF-1Eb and placental vascular pathology. Although IGF-1 signaling has been implicated in endothelial function and vascular homeostasis in previous studies, the present study did not assess vascular function, signaling pathway activity, or the mechanisms underlying the observed immunoreactivity. Therefore, any potential role of IGF-1Eb in vascular adaptation or response to placental hypoperfusion remains speculative and requires direct functional investigation. Chronic ischemic and oxidative stress associated with MVM could plausibly stimulate local IGF-1Eb synthesis, retention, or altered processing through HIF-mediated and post-transcriptional mechanisms. The significant association between endothelial immunopositivity and vascular pathology observed in the present study supports an association between IGF-1Eb immunoreactivity and placental vascular pathology; however, whether IGF-1Eb directly contributes to vascular adaptation cannot be determined from the present observational data. It is important to distinguish established biological mechanisms from interpretations that can be drawn from the present findings. IGF-1 signaling has been linked in previous studies to pathways involved in placental growth, trophoblast function, cellular metabolism, and vascular biology, including PI3K/AKT and mTOR-related signaling and responses to hypoxic stress. However, these pathways were not directly investigated in the present study. Therefore, the observed differences in IGF-1Eb protein immunoreactivity cannot be interpreted as evidence of altered PI3K/AKT, mTOR, hypoxia-responsive signaling, trophoblast function, or vascular remodeling. Rather, our findings demonstrate compartment-specific differences in IGF-1Eb protein immunoreactivity that are associated with FGR and selected histopathological features. Whether these differences represent a causal mechanism, a compensatory response, or a secondary consequence of placental pathology remains unknown and requires direct functional investigation. These findings also complement previous work from our group examining placental IGF-1Ea immunoreactivity in FGR pregnancies. Prior analysis demonstrated altered IGF-1Ea immunolocalization associated with placental vascular pathology and maternal vascular malperfusion [13]. Interestingly, IGF-1Eb displayed both overlapping and divergent patterns of immunoreactivity. Similarly to IGF-1Ea, increased IGF-1Eb immunoreactivity correlated with vascular dysfunction and histopathological abnormalities. However, IGF-1Eb demonstrated particularly prominent endothelial localization, suggesting potentially distinct biological functions among splice variants. Such observations support the emerging concept that alternative IGF-1 transcripts are not merely redundant by-products of gene expression but may instead mediate complementary and highly specialized actions within placental tissue.

Several strengths of this study should be acknowledged. Placental lesions were classified according to the Amsterdam consensus criteria [18], ensuring standardized and internationally accepted pathological assessment. Furthermore, the integration of quantitative mRNA analysis with immunohistochemical assessment permitted evaluation of both transcriptional changes and compartment-specific patterns of protein localization and immunoreactivity, providing a more comprehensive characterization of IGF-1Eb regulation than either approach alone. Detailed clinicopathological and histopathological correlations further strengthened the interpretation of the findings and provided relevant pathological and clinical context.

Several limitations should be considered when interpreting the present findings. First, the control group was relatively small and imbalanced compared with the FGR group (15 AGA vs. 47 FGR placentas). This imbalance primarily affects the precision of the estimates rather than their validity. With only 15 controls, a single discordant observation changes the estimated control proportion by 6.7 percentage points, contributing to the wide confidence intervals observed for some odds ratios. The upper limits of these intervals should therefore not be interpreted as plausible estimates of effect magnitude. Absolute risk differences, which are more informative and stable at these sample sizes, were consequently reported alongside ratio measures. A more balanced distribution of placentas would have resulted in narrower confidence intervals and greater statistical precision.

The RT-qPCR analysis was also limited by the absence of an a priori sample size calculation and by the relatively high variability in transcript measurements. Under these conditions, the analysis was sufficiently powered primarily to detect large differences in mRNA abundance, approximately ten-fold or greater. Thus, the lack of a statistically significant difference in transcript levels should be interpreted as insufficient evidence to demonstrate a difference rather than as evidence that no difference exists. Accordingly, any inference regarding post-transcriptional regulation remains tentative.

The subgroup analyses were exploratory and were not defined a priori. Of the 77 estimable comparisons reported in Table 2, Table 3, Table 4, Table 5 and Table 6, 31 reached nominal significance and 28 of these remained significant after Benjamini–Hochberg correction, consistent with the figures reported in Section 3.3. Several subgroups contained fewer than five placentas per arm, while others could not be estimated because one group contained no observations. These findings should therefore be regarded as hypothesis-generating, and none of the principal conclusions of the study relies on these subgroup analyses.

With regard to immunohistochemical assessment, although tissue preparations were independently evaluated by two experienced pathologists, only the final consensus scores were retained. Consequently, interobserver agreement could not be quantified using Cohen’s kappa or an equivalent statistic. Moreover, the semi-quantitative nature of immunohistochemical scoring inherently introduces a degree of observer subjectivity. Future studies should retain individual observer scores and formally assess interobserver reproducibility using an appropriate agreement coefficient.

The clinical characterization of the FGR cohort represents another important limitation. Doppler parameters and specific markers of placental dysfunction were not systematically collected in the original cohort, preventing retrospective classification according to contemporary multidimensional FGR criteria. In addition, although an EFW below the 10th percentile is commonly used to identify growth restriction, an EFW below the fifth percentile was deliberately selected as the inclusion threshold to enrich the cohort for more severe growth restriction. The findings should therefore be interpreted within the context of this more stringent ultrasound-based definition.

Furthermore, the placental samples were derived from a cohort previously investigated for IGF-1Ea mRNA expression and immunoreactivity [13], and complete patient-level information regarding preeclampsia was not available for all archived cases. Consequently, the exclusion of preeclampsia could not be retrospectively verified for every pregnancy, and the cohort cannot be considered a strictly defined population of idiopathic FGR. This is relevant because preeclampsia may independently contribute to the molecular and histopathological abnormalities observed in FGR placentas. Prospective studies applying predefined exclusion criteria for preeclampsia and contemporary diagnostic criteria for FGR are therefore needed to determine whether the observed IGF-1Eb alterations are specific to idiopathic FGR. Because preeclampsia status could not be established for every case, it could not be entered into the multivariable models, and the adjusted estimates therefore reflect adjustment for the available covariates only. The present data consequently cannot determine whether the IGF-1Eb pattern observed here is specific to FGR, reflects preeclampsia-associated placental dysfunction, or represents a placental phenotype shared by both.

Another consideration is the difference in gestational age at delivery between the FGR and AGA groups. The AGA pregnancies were predominantly uncomplicated and reached term, whereas FGR may necessitate preterm delivery because of fetal or placental compromise. As a result, gestational age represents a potential confounder in comparisons of placental IGF-1Eb expression. The current design cannot determine whether differences observed in preterm FGR placentas are attributable specifically to FGR or partly reflect gestational-age-related changes in placental maturation. Because appropriately grown preterm controls were not available, gestational-age-matched comparisons could not be performed. Future prospective studies should therefore include AGA controls matched for gestational age, particularly among preterm deliveries, to distinguish disease-related alterations from developmental changes in placental IGF-1Eb immunoreactivity. This limitation bears directly on the interpretation of the adjusted models. Because no preterm stratum contains an AGA placenta, Model 1 adjusts for gestational age in the absence of any overlap between the groups below 37 weeks, and gestational age at delivery is itself influenced in FGR pregnancies by disease severity and by the timing of intervention. The adjusted estimates should therefore be understood as reducing rather than removing gestational-age-related confounding, and the possibility that differences in preterm placentas partly reflect placental maturation applies equally to the adjusted and the unadjusted comparisons.

Finally, the observational and cross-sectional design precludes conclusions regarding causality, and residual confounding cannot be excluded despite multivariable adjustment. In addition, no functional experiments were performed to determine the biological consequences of altered IGF-1Eb expression or immunoreactivity. The observed differences may therefore represent causal, compensatory, protective, or secondary responses to placental pathology. Functional studies will be required to establish the specific role of IGF-1Eb in placental tissue and to clarify the mechanisms underlying its association with FGR and related histopathological features.

Future research should build on these findings by integrating adequately powered, gestational-age-matched cohorts with molecular and functional approaches to determine whether altered IGF-1Eb immunoreactivity represents a disease-specific feature of placental dysfunction and to elucidate its biological role in idiopathic FGR. Experimental studies using trophoblast and endothelial cell models, together with analyses of hypoxia-responsive signaling and splice-variant-specific activity, may help determine whether IGF-1Eb directly modulates trophoblast survival, angiogenesis, or nutrient transport. Longitudinal and early gestational studies may further clarify temporal regulation of this isoform and its potential involvement in placental development before establishment of overt growth restriction.

## 5. Conclusions

Placental IGF-1Eb protein immunoreactivity, but not IGF-1Eb mRNA expression, differed between FGR and AGA pregnancies in selected placental compartments, and these differences persisted after adjustment for the available covariates. The observed differences in immunoreactivity, particularly in the perivillous syncytiotrophoblast and the endothelium of maternal and fetal vessels, were associated with histopathological features of placental pathology, including maternal vascular malperfusion (MVM), although this association was not maintained once study group was taken into account. Overall, the findings indicate that IGF-1Eb immunoreactivity differs between FGR and AGA placentas in selected placental compartments, with associations persisting after adjustment for some available covariates.

These findings suggest compartment-specific alterations in placental IGF-1Eb immunoreactivity in pregnancies complicated by FGR, but they do not establish disease specificity. Because preeclampsia status could not be ascertained for every case and appropriately grown preterm controls were unavailable, the study cannot determine whether the observed pattern is specific to FGR, reflects preeclampsia-associated placental dysfunction, or represents a placental phenotype shared by both, nor whether differences in preterm placentas partly reflect placental maturation. Given the observational and cross-sectional design of this study, these associations should not be interpreted as evidence of causality or as establishing a specific mechanistic role for IGF-1Eb in placental dysfunction.

Larger prospective studies incorporating gestational-age-matched controls and predefined exclusion criteria for preeclampsia, together with functional validation, are warranted to confirm these findings and clarify their biological significance. Further investigation is also required to determine whether IGF-1Eb may represent a candidate biomarker of placental dysfunction and whether such a potential role has clinical relevance.

## Figures and Tables

**Table 1:** Maternal and pregnancy characteristics of the pregnancies included in the placental mRNA analysis.

Characteristics	AGA Placenta (*n* = 13)	FGR Placenta (*n* = 15)
Maternal age, years		
<40	12 (92.3%)	10 (66.7%)
≥40	1 (7.7%)	5 (33.3%)
Gestational age at delivery, weeks		
<37 weeks	0 (0%)	5 (33.3%)
>37 weeks	13 (100%)	10 (66.7%)
Neonatal birth weight, g		
<2500	0 (0%)	9 (60.0%)
>2500	13 (100%)	6 (40.0%)
Placental weight, g		
<400	0 (0%)	3 (20.0%)
>400	13 (100%)	12 (80.0%)
Maternal BMI, kg/m^2^		
<30	11 (84.6%)	9 (60.0%)
>30	2 (15.4%)	6 (40.0%)
Fetal sex		
Male (%)	6 (46.2%)	7 (46.7%)
Female (%)	7 (53.8%)	8 (53.3%)

Values are presented as *n* (%). Table 1 summarizes the clinical characteristics corresponding to the pregnancies included in the placental mRNA analysis. The underlying categorical distributions are derived from the previously reported molecular-analysis cohort [13].

**Table 2:** IGF-1Eb immunoreactivity scores in the perivillous syncytiotrophoblasts.

Clinicopathological Parameters	AGA (%)			FGR (%)			*p*-Value (*q*-Value)
	Negative	Moderate	High	Negative	Moderate	High	
Total	14 (93.3)	1 (6.7)	0 (0.0)	22 (46.8)	21 (44.7)	4 (8.5)	0.006 (0.018)
Age, years							
<40	13 (92.9)	1 (7.1)	0 (0.0)	18 (48.6)	17 (45.9)	2 (5.4)	0.015 (0.043)
≥40	1 (100.0)	0 (0.0)	0 (0.0)	2 (33.3)	2 (33.3)	2 (33.3)	0.459 (0.599)
Neonatal birth weight, g							
<2500	0 (0.0)	0 (0.0)	0 (0.0)	11 (37.9)	15 (51.7)	3 (10.3)	-
≥2500	14 (93.3)	1 (6.7)	0 (0.0)	5 (50.0)	4 (40.0)	1 (10.0)	0.043 (0.107)
Placental weight, g							
<400	0 (0.0)	0 (0.0)	0 (0.0)	17 (54.8)	12 (38.7)	2 (6.5)	-
≥400	14 (93.3)	1 (6.7)	0 (0.0)	5 (31.2)	9 (56.2)	2 (12.5)	0.002 (0.008)
BMI, Kg/m^2^							
<30	11 (91.7)	1 (8.3)	0 (0.0)	2 (18.2)	7 (63.6)	2 (18.2)	0.002 (0.008)
≥30	3 (100.0)	0 (0.0)	0 (0.0)	1 (16.7)	4 (66.7)	1 (16.7)	0.060 (0.127)
Fetal sex, %							
Male	6 (85.7)	1 (14.3)	0 (0.0)	3 (37.5)	4 (50.0)	1 (12.5)	0.153 (0.274)
Female	8 (100.0)	0 (0.0)	0 (0.0)	5 (25.0)	13 (65.0)	2 (10.0)	0.002 (0.008)
Histopathology, %							
MVM	11 (91.7)	1 (8.3)	0 (0.0)	15 (42.9)	16 (45.7)	4 (11.4)	0.015 (0.043)
MPFD	0 (0.0)	0 (0.0)	0 (0.0)	3 (75.0)	1 (25.0)	0 (0.0)	-
FVM	0 (0.0)	0 (0.0)	0 (0.0)	1 (100.0)	0 (0.0)	0 (0.0)	-
VUE	1 (100.0)	0 (0.0)	0 (0.0)	4 (80.0)	1 (20.0)	0 (0.0)	-
DVM	2 (100.0)	0 (0.0)	0 (0.0)	2 (40.0)	3 (60.0)	0 (0.0)	-
Other	0 (0.0)	0 (0.0)	0 (0.0)	3 (75.0)	1 (25.0)	0 (0.0)	-

Values in parentheses in the *p*-value column are Benjamini–Hochberg-adjusted q-values, computed across all 77 estimable comparisons reported in Table 2, Table 3, Table 4, Table 5 and Table 6 (false discovery rate 5%). A dash indicates a comparison that is not estimable because one group contained no placentas in that stratum. Strata containing fewer than five placentas in either group (i.e., maternal age ≥ 40 years, MPFD, FVM, VUE, DVM and other lesions) are exploratory and are reported for completeness only.

**Table 3:** IGF-1Eb immunoreactivity intensity in the perivillous syncytiotrophoblast of AGA and FGR human placentas obtained from third-trimester pregnancies.

Clinicopathological Parameters	AGA (%)			FGR (%)			*p*-Value (*q*-Value)
	Low	Moderate	Strong	Low	Moderate	Strong	
Total	14 (93.3)	1 (6.7)	0 (0.0)	36 (76.6)	9 (19.1)	2(4.3)	0.381 (0.554)
Age, years							
<40	13 (92.9)	1 (7.1)	0 (0.0)	29 (78.4)	6 (16.2)	2 (5.4)	0.444 (0.599)
≥40	1 (100.0)	0 (0.0)	0 (0.0)	4 (66.7)	2 (33.3)	0 (0.0)	0.495 (0.615)
Gestational age, weeks							
<37	0 (0.0)	0 (0.0)	0 (0.0)	18 (69.2)	6 (23.1)	2 (7.7)	-
≥37	14 (93.3)	1 (6/7)	0 (0.0)	18 (85.7)	3 (14.3)	0 (0.0)	0.626 (0.699)
Neonatal weight, g							
<2500	0 (0.0)	0 (0.0)	0 (0.0)	21 (72.4)	6 (20.7)	2 (6.9)	-
≥2500	14 (93.3)	1 (6.7)	0 (0.0)	8 (80.0)	2 (20.0)	0 (0.0)	1.000 (1.000)
Placental weight, g							
<400	0 (0.0)	0 (0.0)	0 (0.0)	22 (71.0)	7 (22.6)	2 (6.5)	-
≥400	14 (93.3)	1 (6.7)	0 (0.0)	14 (87.5)	2 (12.5)	0 (0.0)	1.000 (1.000)
BMI, Kg/m^2^							
<30	11 (91.7)	1 (8.3)	0 (0.0)	9 (81.8)	2 (18.2)	0 (0.0)	0.484 (0.611)
≥30	3 (100.0)	0 (0.0)	0 (0.0)	5 (83.3)	1 (16.7)	0 (0.0)	0.453 (0.599)
Fetal sex, %							
Male	6 (85.7)	1 (14.3)	0 (0.0)	6 (75.0)	2 (25.0)	0 (0.0)	0.605 (0.695)
Female	8 (100.0)	0 (0.0)	0 (0.0)	15 (75.0)	3 (15.0)	0 (0.0)	0.296 (0.456)
Histopathology, %							
MVM	11 (91.7)	1 (8.3)	0 (0.0)	27 (77.1)	6 (17.1)	2 (5.7)	0.553 (0.655)
MPFD	0 (0.0)	0 (0.0)	0 (0.0)	2 (50.0)	2 (50.0)	0 (0.0)	-
FVM	0 (0.0)	0 (0.0)	0 (0.0)	1 (100.0)	0 (0.0)	0 (0.0)	-
VUE	1 (100.0)	0 (0.0)	0 (0.0)	4 (80.0)	1 (20.0)	0 (0.0)	-
DVM	2 (100.0)	0 (0.0)	0 (0.0)	4 (80.0)	1 (20.0)	0 (0.0)	-

Values in parentheses in the *p*-value column are Benjamini–Hochberg-adjusted q-values, computed across all 77 estimable comparisons reported in Table 2, Table 3, Table 4, Table 5 and Table 6 (false discovery rate 5%). A dash indicates a comparison that is not estimable because one group contained no placentas in that stratum. Strata containing fewer than five placentas in either group (i.e., maternal age ≥ 40 years, MPFD, FVM, VUE, DVM and other lesions) are exploratory and are reported for completeness only.

**Table 4:** IGF-1Eb immunoreactivity in extravillous trophoblasts of AGA and FGR human third-trimester placentas.

Clinicopathological Parameters	AGA (%)		FGR (%)		*p*-Value (*q*-Value)
	Negative	Positive	Negative	Positive	
Total	4 (26.7)	11 (73.3)	18 (38.3)	29 (61.7)	0.412 (0.587)
Age, years					
<40	4 (28.6)	10 (71.4)	14 (37.8)	23 (62.2)	0.537 (0.646)
≥40	0 (0.0)	1 (100.0)	3 (50.0)	3 (50.0)	0.350 (0.518)
Gestational age, weeks					
<37	0 (0.0)	0 (0.0)	7 (26.9)	19 (73.1)	-
≥37	4 (26.7)	11 (73.3)	11 (52.4)	10(47.6)	0.123 (226)
Neonate weight, g					
<2500	0 (0.0)	0 (0.0)	13 (44.8)	16 (55.2)	-
≥2500	4 (26.7)	11 (73.3)	5 (50.0)	5 (50.0)	0.234 (0.400)
Placental weight, g					
<400	0 (0.0)	0 (0.0)	13 (41.9)	18 (58.1)	-
≥400	4 (26.7)	11 (73.3)	5 (31.3)	11 (68.8)	0.779 (0.857)
BMI, Kg/m^2^					
<30	4 (33.3)	8 (66.7)	4 (36.4)	7 (63.6)	0.879 (0.940)
≥30	0 (0.0)	3 (100.0)	1 (16.7)	5 (83.3)	0.453 (0.599)
Fetal sex, %					
Male	1 (14.3)	6 (85.7)	2 (25.0)	6 (75.0)	0.605 (0.695)
Female	3 (37.5)	5 (62.5)	8 (40.0)	12 (60.0)	0.903 (0.952)
Histopathology, %					
MVM	2 (16.7)	10 (83.3)	13 (34.3)	23 (65.7)	0.249 (0.417)
MPFD	0 (0.0)	0 (0.0)	3 (75)	1 (25)	-
FVM	0 (0.0)	0 (0.0)	1 (100.0)	0 (0.0)	-
VUE	0 (0.0)	1 (100.0)	3 (60.0)	2 (40.0)	0.273 (0.447)
DVM	2 (100.0)	0 (0.0)	1 (20.0)	4 (80.0)	0.053 (0.124)
Other	0 (0.0)	0 (0.0)	2 (50.0)	2 (50.0)	-

Values in parentheses in the *p*-value column are Benjamini–Hochberg-adjusted q-values, computed across all 77 estimable comparisons reported in Table 2, Table 3, Table 4, Table 5 and Table 6 (false discovery rate 5%). A dash indicates a comparison that is not estimable because one group contained no placentas in that stratum. Strata containing fewer than five placentas in either group (i.e., maternal age ≥ 40 years, MPFD, FVM, VUE, DVM and other lesions) are exploratory and are reported for completeness only.

**Table 5:** IGF-1Eb immunohistochemical staining intensity in extravillous trophoblasts of third-trimester human placentas.

Clinicopathological Parameters	AGA (%)			FGR (%)			*p*-Value (*q*-Value)
	Weak	Moderate	Strong	Weak	Moderate	Strong	
Total	11 (73.3)	4 (26.7)	0 (0.0)	30 (65.2)	9 (19.6)	7 (15.2)	0.287 (0.456)
Age, years							
<40	10 (71.4)	4 (28.6)	0 (0.0)	19 (70.4)	4 (14.8)	4 (14.8)	0.292 (0.456)
≥40	1 (100.0)	0 (0.0)	0 (0.0)	10 (6.7)	4 (26.7)	1 (6.7)	1.000 (1.000)
Gestational age, weeks							
<37	0 (0.0)	0 (0.0)	0 (0.0)	13 (52.0)	6 (24.0)	6 (24.0)	-
≥37	1 (73.3)	4 (26.7)	0 (0.0)	17 (81.0)	3 (14.3)	1 (4.8)	0.809 (0.877)
Neonate weight, g							
<2500	0 (0.0)	0 (0.0)	0 (0.0)	16 (57.2)	6 (21.4)	6 (21.4)	-
≥2500	11 (73.3)	4 (26.7)	0 (0.0)	9 (90.0)	1 (10.0)	0 (0.0)	0.615 (0.696)
Placental weight, g							
<400	0 (0.0)	0 (0.0)	0 (0.0)	15 (50.0)	8 (26.7)	7 (23.3)	-
≥400	11 (73.3)	4 (26.7)	0 (0.0)	15 (93.8)	1 (6.2)	0 (0.0)	0.172 (0.301)
BMI, Kg/m^2^							
<30	0 (0.0)	0 (0.0)	0 (0.0)	22 (71.0)	2 (22.6)	2 (6.5)	-
≥30	14 (93.3)	1 (6.7)	0 (0.0)	14 (87.5)	2 (12.5)	0 (0.0)	1.000 (1.000)
Fetal sex, %							
Male	11 (91.7)	1 (8.3)	0 (0.0)	9 (81.8)	2 (18.2)	0 (0.0)	0.484 (0.611)
Female	3 (100.0)	0 (0.0)	0 (0.0)	5 (83.3)	1 (16.7)	0 (0.0)	0.453 (0.599)
Histopathology, %							
MVM	10 (83.3)	2 (16.7)	0 (0.0)	25 (73.5)	4 (11.8)	5 (14.7)	0.523 (0.639)
MPFD	0 (0.0)	0 (0.0)	0 (0.0)	2 (50.0)	2 (50.0)	0 (0.0)	-
FVM	0 (0.0)	0 (0.0)	0 (0.0)	1 (100.0)	0 (0.0)	0 (0.0)	-
VUE	1 (100.0)	0 (0.0)	0 (0.0)	2 (40.0)	3 (60.0)	0 (0.0)	-
DVM	0 (0.0)	2 (100.0)	0 (0.0)	2 (40.0)	2 (40.0)	1 (20.0)	-
Other	0 (0.0)	0 (0.0)	0 (0.0)	2 (50.0)	1 (25.0)	1 (25.0)	-

Values in parentheses in the *p*-value column are Benjamini–Hochberg-adjusted q-values, computed across all 77 estimable comparisons reported in Table 2, Table 3, Table 4, Table 5 and Table 6 (false discovery rate 5%). A dash indicates a comparison that is not estimable because one group contained no placentas in that stratum. Strata containing fewer than five placentas in either group (i.e., maternal age ≥ 40 years, MPFD, FVM, VUE, DVM and other lesions) are exploratory and are reported for completeness only.

**Table 6:** IGF-1Eb immunoreactivity in maternal and fetal placental vascular endothelium in third-trimester pregnancies.

Clinicopathological Parameters	Maternal Vessels			Fetal Stem–Intermediate Vessels			Fetal Distal–Peripheral Vessels		
	AGA	FGR	*p*-Value	AGA	FGR	*p*-Value	AGA	FGR	*p*-Value (*q*-Value)
Total	2 (13.3)	35 (74.5)	<0.001	3 (20.0)	36 (76.6)	<0.001	2 (13.3)	20 (43.5)	0.061 (0.127)
Age									
<40	2 (14.3)	27 (73.0)	<0.001	3 (21.4)	28 (75.7)	<0.001	2 (14.3)	16 (43.2)	0.053 (0.124)
≥40	0 (0.0)	5 (83.3)	0.088	0 (0.0)	5 (83.3)	0.088	0 (0.0)	3 (50.0)	0.350 (0.518)
Gestational age, weeks									
<37	0 (0.0)	18 (69.2)	-	0 (0.0)	19 (73.1)	-	0 (0.0)	10 (38.5)	-
≥37	2 (13.3)	17 (81.0)	<0.001	3 (20.0)	17 (81.0)	0.001	2 (13.3)	10 (50.0)	0.034 (0.087)
Birth weight									
<2500	0 (0.0)	25 (86.2)	-	0 (0.0)	26 (89.7)	-	0 (0.0)	13 (46.4)	-
≥2500	2 (13.3)	8 (80.0)	0.002	3 (20.0)	8 (80.0)	0.005	2 (13.3)	6 (60.0)	0.028 (0.074)
Placental weight, g									
<400	0 (0.0)	23 (74.2)	-	0 (0.0)	22 (71.0)	-	0 (0.0)	8 (26.7)	-
≥400	2 (13.3)	12 (75.0)	0.001	3 (20.0)	14 (87.5)	<0.001	2 (13.3)	12 (75.0)	0.001 (0.005)
BMI, Kg/m^2^									
<30	2 (16.7)	11 (100.0)	<0.001	3 (25.9)	11 (100.0)	<0.001	2 (16.7)	11 (100.0)	<0.001 (0.004)
≥30	0 (0.0)	4 (66.7)	0.058	0 (0.0)	5 (83.3)	0.018	0 (0.0)	4 (66.6)	0.058 (0.127)
Fetal sex, %									
Male	1 (14.3)	7 (87.5)	0.005	2 (28.6)	8 (100.0)	0.003	2 (28.6)	6 (75.0)	0.072 (0.146)
Female	1 (12.5)	17 (85.0)	<0.01	1 (12.5)	17 (85.0)	<0.001	0 (0.0)	12 (60.0)	0.004 (0.015)
Histopathology									
MVM	2 (16.7)	25 (71.4)	0.002	3 (25.0)	26 (74.3)	0.005	2 (16.7)	17 (50.0)	0.086 (0.165)
MPFD	0 (0.0)	2 (66.7)	-	0 (0.0)	2 (66.7)	-	0 (0.0)	0 (0.0)	-
FVM	0 (0.0)	1 (100.0)	-	0 (0.0)	1 (100.0)	-	0 (0.0)	0 (0.0)	-
VUE	0 (0.0)	4 (80.0)	-	0 (0.0)	3 (60.0)	-	0 (0.0)	0 (0.0)	-
DVM	0 (0.0)	3 (60.0)	-	0 (0.0)	3 (60.0)	-	0 (0.0)	2 (40.0)	-
Other	0 (0.0)	3 (75.0)	-	0 (0.0)	3 (75.0)	-	0 (0.0)	1 (25.0)	-

Values in parentheses in the *p*-value column are Benjamini–Hochberg-adjusted q-values, computed across all 77 estimable comparisons reported in Table 2, Table 3, Table 4, Table 5 and Table 6 (false discovery rate 5%). A dash indicates a comparison that is not estimable because one group contained no placentas in that stratum. Strata containing fewer than five placentas in either group (i.e., maternal age ≥ 40 years, MPFD, FVM, VUE, DVM and other lesions) are exploratory and are reported for completeness only.

**Table 7:** Effect sizes for the overall comparison of IGF-1Eb immunoreactivity between FGR and AGA placentas Odds ratios are conditional maximum-likelihood estimates with exact 95% confidence intervals; risk differences use Newcombe score intervals.

Compartment	FGR n/N (%)	AGA n/N (%)	Odds Ratio (95% CI)	Risk Difference, % (95% CI)	Cramér’s V
Perivillous syncytiotrophoblast, positive immunoreactivity	25/47 (53.2)	1/15 (6.7)	15.34 (2.02–698.94)	46.5 (19.5 to 61.1)	0.40
Perivillous syncytiotrophoblast, moderate or strong intensity	11/47 (23.4)	1/15 (6.7)	4.20 (0.52–197.07)	16.7 (−8.4 to 31.6)	0.18
Extravillous trophoblast, positive immunoreactivity	29/47 (61.7)	11/15 (73.3)	0.59 (0.12–2.40)	−11.6 (−32.9 to 16.6)	0.10
Extravillous trophoblast, moderate or strong intensity	25/47 (53.2)	11/15 (73.3)	0.42 (0.08–1.68)	−20.1 (−41.2 to 8.5)	0.17
Maternal decidual vessel endothelium, positive immunoreactivity	35/47 (74.5)	2/15 (13.3)	17.91 (3.36–186.09)	61.1 (32.9 to 75.2)	0.53
Fetal stem–intermediate vessel endothelium, positive immunoreactivity	36/47 (76.6)	3/15 (20.0)	12.43 (2.72–81.07)	56.6 (27.9 to 72.8)	0.50
Fetal distal–peripheral vessel endothelium, positive immunoreactivity	20/46 (43.5)	2/15 (13.3)	4.89 (0.94–49.53)	30.1 (2.2 to 47.3)	0.27

Confidence intervals for the odds ratios are wide because the control group comprises 15 placentas; the upper limits should not be interpreted as plausible effect sizes. Absolute risk differences are better determined at these sample sizes and are the preferred summary of each contrast. n/N denotes the number of placentas with positive immunoreactivity out of the number assessed.

**Table 8:** Multivariable association between FGR and IGF-1Eb immunoreactivity. Values are adjusted odds ratios for FGR versus AGA with 95% confidence intervals, estimated by Firth penalized logistic regression.

Compartment	Model 1 (n = 62)	Model 2 (Sensitivity Analysis, n = 40)	Model 3 (n = 62)
Perivillous syncytiotrophoblast, positive immunoreactivity	7.36 (1.38–76.01), *p* = 0.017	19.57 (2.88–238.84), *p* = 0.002	7.65 (1.43–79.23), *p* = 0.015
Perivillous syncytiotrophoblast, moderate or strong intensity	1.29 (0.20–14.42), *p* = 0.800	0.93 (0.09–12.22), *p* = 0.950	1.31 (0.20–14.51), *p* = 0.789
Extravillous trophoblast, positive immunoreactivity	0.36 (0.08–1.43), *p* = 0.149	0.64 (0.12–3.32), *p* = 0.595	0.36 (0.08–1.47), *p* = 0.158
Extravillous trophoblast, moderate or strong intensity	0.32 (0.07–1.23), *p* = 0.099	0.35 (0.06–1.72), *p* = 0.199	0.32 (0.07–1.25), *p* = 0.103
Maternal decidual vessel endothelium, positive immunoreactivity	14.87 (3.19–96.63), *p* < 0.001	33.79 (4.45–471.54), *p* < 0.001	14.19 (3.07–91.14), *p* < 0.001
Fetal stem–intermediate vessel endothelium, positive immunoreactivity	10.68 (2.47–57.00), *p* = 0.001	47.92 (5.19–1064), *p* < 0.001	10.24 (2.40–53.79), *p* = 0.001
Fetal distal–peripheral vessel endothelium, positive immunoreactivity	5.73 (1.27–35.63), *p* = 0.022	35.67 (4.91–495.79), *p* < 0.001	6.24 (1.35–39.95), *p* = 0.018

## Data Availability

The datasets used and/or analyzed during this study are available from the corresponding author on reasonable request.
